# Plasminogen Activator Inhibitor-1 Controls Vascular Integrity by Regulating VE-Cadherin Trafficking

**DOI:** 10.1371/journal.pone.0145684

**Published:** 2015-12-29

**Authors:** Anna E. Daniel, Ilse Timmerman, Igor Kovacevic, Peter L. Hordijk, Luc Adriaanse, Ilkka Paatero, Heinz-Georg Belting, Jaap D. van Buul

**Affiliations:** 1 Department of Molecular Cell Biology, Sanquin Research and Landsteiner Laboratory, Academic Medical Center, University of Amsterdam, Amsterdam, the Netherlands; 2 Department of Cell Biology, Biozentrum der Universität Basel, Basel, Switzerland; University of Illinois at Chicago, UNITED STATES

## Abstract

**Background:**

Plasminogen activator inhibitor-1 (PAI-1), a serine protease inhibitor, is expressed and secreted by endothelial cells. Patients with PAI-1 deficiency show a mild to moderate bleeding diathesis, which has been exclusively ascribed to the function of PAI-1 in down-regulating fibrinolysis. We tested the hypothesis that PAI-1 function plays a direct role in controlling vascular integrity and permeability by keeping endothelial cell-cell junctions intact.

**Methodology/Principal Findings:**

We utilized PAI-039, a specific small molecule inhibitor of PAI-1, to investigate the role of PAI-1 in protecting endothelial integrity. *In vivo* inhibition of PAI-1 resulted in vascular leakage from intersegmental vessels and in the hindbrain of zebrafish embryos. In addition PAI-1 inhibition in human umbilical vein endothelial cell (HUVEC) monolayers leads to a marked decrease of transendothelial resistance and disrupted endothelial junctions. The total level of the endothelial junction regulator VE-cadherin was reduced, whereas surface VE-cadherin expression was unaltered. Moreover, PAI-1 inhibition reduced the shedding of VE-cadherin. Finally, we detected an accumulation of VE-cadherin at the Golgi apparatus.

**Conclusions/Significance:**

Our findings indicate that PAI-1 function is important for the maintenance of endothelial monolayer and vascular integrity by controlling VE-cadherin trafficking to and from the plasma membrane. Our data further suggest that therapies using PAI-1 antagonists like PAI-039 ought to be used with caution to avoid disruption of the vessel wall.

## Introduction

Endothelia line all blood vessels and form a barrier between the circulation and surrounding tissues. The barrier function is maintained and regulated by the adherens junctions that connect neighboring cells. The most important cell adhesion protein in endothelial adherens junctions is VE-cadherin (Vascular Endothelial-cadherin), which ensures that endothelial cells stay connected and restrains the leakage from blood vessels [1]. VE-cadherin interacts with p120 catenin, which prevents internalization of VE-cadherin [2,3], and with β- and α-catenin which anchor VE-cadherin to the actin cytoskeleton [4,5]. Disruption of VE-cadherin-based junctions, e.g. in response to inflammatory mediators like TNFα, leads to a loss of endothelial integrity which is accompanied by increased endothelial permeability [6,7].

PAI-1 is a serine protease, which is expressed and secreted by endothelial cells, hepatocytes, adipocytes, megakaryocytes and neuronal cells [8]. PAI-1 has an important role in keeping thrombus formation and fibrinolysis in balance. As an inhibitor of urokinase (uPA) and tissue plasminogen activator (tPA) PAI-1 prevents the formation of plasmin from plasminogen and therefore inhibits the (excessive) degradation of fibrin. Apart from the role of PAI-1 as an inhibitor of fibrinolytic activity, other PAI-1 functions for cell adhesion to extracellular matrix, tissue remodeling, migration, proliferation and apoptosis have been described [9–14]. Patients with PAI-1 deficiency have an increased risk for hemorrhaging after surgery, menorrhagia and epistaxis [15]. This phenotype has to date been solely ascribed to the function of PAI-1 as an inhibitor of fibrinolysis.

We show here that PAI-1 function is necessary for the maintenance of endothelial monolayer integrity. Further we show that PAI-1 inhibition with the small molecule inhibitors PAI-039 and TM5275 disrupts endothelial cell-cell junctions and causes a loss of transendothelial resistance of HUVEC monolayers and increased vascular permeability in zebrafish. We link PAI-1 function to the maintenance of endothelial cell barriers both *in vivo* in zebrafish and *in vitro* with HUVEC cultures. This indicates that therapeutic administration of PAI-1 antagonists like PAI-039 ought to be used with caution to avoid side effects like leakage of the vessel wall.

## Materials and Methods

### Materials

HUVEC pools were purchased from Invitrogen (Breda, The Netherlands). EGM-2 medium and SingleQuots^™^ for HUVEC cell culture were purchased from Lonza (Verviers, Belgium). PAI-1 inhibitors PAI-039 (tiplaxtinin) and TM5275 were purchased from Axon Medchem BV (Groningen, The Netherlands) and diluted in DMSO (referred to as solvent). PAI-1 rabbit polyclonal antibody was a kind gift from Dr. Sacha Zeerleder. VE-cadherin monoclonal mouse antibody, clone BV6, was purchased from Millipore (Amsterdam, The Netherlands) and actin [AC-40] monoclonal mouse antibody was purchased from Sigma (Zwijndrecht, The Netherlands). GM130 rabbit monoclonal antibody was from Cell Signaling (Leiden, The Netherlands). Goat polyclonal anti-VE-cadherin [C-19] and rabbit polyclonal anti-α-catenin were purchased from Santa Cruz Biotechnology (Heidelberg, Germany). Mouse monoclonal antibodies directed against mouse monoclonal PECAM-1 AF488 [WM59], VE-cadherin AF 647 [55-7H1], IgG1 AF674, β-catenin [14] and p120 catenin [98/pp120] were purchased from BD Biosciences (Amsterdam, The Netherlands). Texas Red-X Phalloidin, chicken anti-goat AF647, chicken anti-rabbit AF488, chicken anti-rabbit AF594, chicken anti-mouse AF488 and IgG1 AF488 were from Invitrogen (Breda, The Netherlands).

### Vascular leakage in zebrafish

Adult zebrafish (*Danio rerio*) of transgenic strain Tg(kdrl:EGFP^s843^) [16] were kept using standard procedures [17] and were cultured at 28°C in E3-medium supplemented with 0.2 mM phenyl-thiourea (Sigma Aldrich) to prevent pigmentation. Embryos were obtained by natural spawning of adult zebrafish in mesh-bottomed breeding-tanks and kept under standard conditions [17]. Maintenance of fish and experimental procedures were carried out at the Biozentrum/Universität Basel according to Swiss national guidelines of animal experimentation (TSchV). Zebrafish lines were bred and maintained under license 1014H issued by the Veterinäramt-Basel-Stadt. Injection of embryos (no longer than 3 days post fertilization; the age of the fish was no more than 3 days, at this stage the embryos are not yet eating on their own) and imaging was performed under anesthesia (40mg tricaine (3-amino benzoic acidethylester)/liter of E3 medium). At the end of the experiments all embryos were immediately euthanized by immersion in ice water (E3) containing 1% hypochlorite solution for at least 5 minutes and independently fed prior to being euthanized. Three days post-fertilization (3dpf), the embryos were treated with 50 μM PAI-039 (as indicated) or with 1% DMSO (solvent). For measurement of vascular leakage from intersomitic vessels we performed micro-angiography according to Isogai et al. [18]. Briefly, embryos were microinjected 2000 kDa tetramethylrhodamine-dextran (TMR-dextran, Molecular Probes Inc.) into the duct of Cuvier (common cardinal vein) with a glass capillary needle and embryos were mounted onto glass-bottom dishes using low-melting point agarose (0.7%) for imaging. Mounted embryos were overlaid with E3-medium supplemented with phenyl-thiourea, Tricaine (0.016%) and treatment with PAI-039 (19 fish) or DMSO (15 fish) was continued. After six hours from the beginning of the inhibitor treatments the tail vasculature dorsal to urogenital opening was imaged using Leica SP5 confocal microscope with 10x (NA 0.3) and 20x (NA 0.7) air objectives. For measurements of leakage to the hindbrain ventricle were performed as described above with following modifications: the 3dpf zebrafish embryos were microinjected as described above with 70 kDa TMR-dextran and treated with 25 μM PAI-039 (25 fish) or solvent control (23 fish) for three to four hours. The inhibitor is dissolved in the drinking solution. Therefore, it is difficult to determine the exact effective dose of the inhibitor. For these reasons, we have doubled the most effective in vitro concentration (25 μM). Then the head of the embryo was imaged using Leica SP5 confocal microscope with 10x (NA 0.3) air objectives. TMR-dextran fluorescence was analyzed utilizing FIJI software. Sum-projections were used for intensity analyses of TMR-dextran intensity located between vessels (extravascular fluorescence) reflecting the relative amount of TMR-dextran leaked out from the vasculature. To confirm comparable illumination of the samples, intensity analyses of intravascular fluorescence of 2000 kDa TMR-dextran within the lumen of dorsal aorta were performed. The level of fluorescence in dorsal aorta was comparable between DMSO (relative fluorescence 1.00 +/- 0.16, n = 15) and PAI-039 treated samples (relative fluorescence 1.04 +/- 0.25, p = 0.747, Mann-Whitney U-test). The level of background signal was measured from the kdrl:EGFP embryos, which were not injected with 2000 kDa TMR-dextran. The relative background signal intensity level of both extravascular and intravascular fluorescence in the TMR channel was low (relative fluorescence 0.024 +/- 0.016, n = 14 and 0.015 +/-0.011, n = 14, respectively). In order to combine data from separate experiments, the raw fluorescence intensity values of the samples within the experiment were normalized with mean value of the control group in the same experiment. Statistical analyses were performed using SPSS and Microsoft Excel.

### Electric cell-substrate impedance sensing (ECIS)

Transendothelial electrical resistance of HUVEC monolayers was measured using ECIS. HUVEC were added at 100,000 cells per well (0.8 cm^2^) of L-cysteine (10 mM, Sigma) and fibronectin (10 μg/mL, Sigma) coated electrode arrays (8W10E PET, Ibidi, Planegg/Martinsried, Germany) in a ECIS Z Theta controller from Applied Biophysics Inc. (Troy, NY, USA). We used a frequency of 4 kHz to continuously measure transendothelial resistance at 37°C at 5% CO_2_. Multiples of samples were used in each experiment. Once the endothelial cells form monolayers the resistance values plateau. We referred to the plateau resistance of endothelial monolayers as “basal resistance”. Inhibitors were added to endothelial monolayers two or more hours after the resistance values reached the plateau. Data were normalized to resistance values one hour before addition of inhibitors.

### Immunofluorescence imaging of HUVEC

HUVEC were cultured on fibronectin-coated glass coverslips until they formed confluent monolayers. For PAI-1 inhibition and to avoid formation of precipitates we removed the conditioned media from the cultured cells and diluted PAI-039 (25 μM, or as indicated) or TM5275 (50 μM, or as indicated) directly in the conditioned media before adding the media back to the cells. As controls we used equivalent amounts of solvent (DMSO, 0.1 or 0.2% as inidicated). We incubated with the inhibitors for up to four hours and then rinsed the cells with phosphate buffered saline (PBS) + 1 mM CaCl_2_ + 0.5 mM MgCl_2_ (PBS++) and fixed with 4% (v/v) paraformaldehyde in PBS++ for 10 minutes. Unspecific antibody binding was blocked by incubating coverslips in 3% (w/v) bovine serum albumin (BSA) in PBS++ for 30 minutes. For staining with antibodies or phalloidin were diluted in 1% BSA in PBS++ and coverslips were incubated with the antibody solutions for 60 minutes. Fluorescent imaging was performed using a confocal laser-scanning microscope (Meta, Carl Zeiss MicroImaging) using a 63x oil lens (NA 1.40).

Image analyses were performed using Image J. To account for the variability of junction width of the cells we took the average junction width at five sites on each cells (S2C Fig). We measured the average junction width of five cells from three different fields of view from both solvent-treated and PAI-039-treated cells from four independent experiments. For analysis of VE-cadherin fluorescence intensity at the Golgi apparatus we first outlined the area of Golgi localization manually on the basis of GM130 stainings. Then we measured size and the intensity of VE-cadherin staining within that area. We analyzed data from ten cells per treatment and experiment from three independent experiments.

### Fluorescence recovery after photobleaching (FRAP)

For FRAP endothelial cells were virally transduced with VE-cadherin-GFP (pLV-CMV-Ires-Puro SIN) [19] and cultured for one day. Then cells were trypsinzed and transferred to Nunc^™^ Lab-Tek^™^ 8-chambered coverglasses (Thermo Scientific, Amsterdam, The Netherlands). After 24 hours 25 μM PAI-039 or 0.1% DMSO (solvent) were added to the culture media. For live-cell imaging Lab-Tek coverglasses were placed in a humidified CO_2_ containing atmosphere (5%) in a heating chamber at 37°C with a a confocal laser-scanning microscope (Meta, Carl Zeiss MicroImaging) and a 63x oil lens (NA 1.40). FRAP imaging was performed after two to three hours of incubation with PAI-039 or DMSO. Photobleaching of 5x5 pixel areas (512x512 resolution) at cell-cell junctions was performed with 100 iterations of 488-nm laser illumination at maximum power (25 mW). Fluorescence recovery was measured by time-lapse imaging. FRAP analysis was performed using Image J, Excel and GraphPad prism. Corrections for cell movements, bleaching and background were made. The data displayed are of average FRAP from five independent experiments with five to ten FRAPs performed per treatment and experiment.

### ELISA

Quantikine human VE-cadherin immunoassay (R&D, Abingdon, UK) was used to measure the release of cleaved VE-cadherin in conditioned media from HUVEC. We cultured HUVEC in supplemented EGM-2 until they reached confluence and then added fresh medium supplemented with either 0.1% DMSO (solvent) or 25 μM PAI-039. After four hours we collected the conditioned media, centrifuged the media to remove cellular debris and snap froze the samples. For the immunoassay samples were measured in duplicate according to the manufacturer’s protocol.

### Fluorescence-activated cell sorting (FACS)

For FACS stainings HUVEC were cultured and treated as described above. HUVEC were rinsed with PBS twice and then detached using Accutase (GE Healthcare, Eindhoven, The Netherlands). Detached cells were resuspended in ice-cold PBS supplemented with 0.5% (w/v) BSA, centrifuged and resuspended in PBS + 0.5% BSA. Cells were incubated with fluorescently labelled antibodies for VE-cadherin or PECAM-1 or isotype matched controls for 30 minutes on ice followed by three washes. Fluorescence was measured using an LSR II (BD, Breda, The Netherlands). FACS analyses were performed with BD FACSDiva (BD, Breda, The Netherlands) and FlowJo (Oregon, USA) software.

### Immunoprecipitation and western blotting

For immunoprecipitation cells were rinsed twice with ice-cold PBS++ and then lysed with ice-cold NP-40 lysis buffer (25 mM Tris, 100 mM NaCl, 10 mM MgCl_2_, 10% (v/v) glycerol and 1% (v/v) Nonidet P-40, pH 7.4) supplemented with protease-inhibitor cocktail tablets (Roche Applied Science). After 10 minutes cells were scraped off dishes and centrifuged at 20,000 xg for 10 minutes at 4°C. The supernatant was incubated with 2 μg of mouse monoclonal VE-cadherin antibody and 50 μl protein G-sepharose beads (GE Healthcare, Dassel, Germany) at 4°C under continuous mixing. The beads were then washed five times with NP-40 lysis buffer (5000 xg, 30 s, 4°C) and boiled (5 min) in sample buffer containing SDS and 4% β-mercapto-ethanol. Samples from immunoprecipitation and total cell lysates were subjected to SDS-PAGE and transferred to 0.2 μm Protran^™^ nitrocellulose membranes (Whatman/GE Healthcare, Dassel, Germany). Membranes were blocked with 5% (w/v) milk powder dissolved in Tris-buffered saline with Tween (TBST, 150 mM NaCl, Tris 10 mM, 0.1% (v/v) Tween20, pH 8.0). The nitrocellulose membranes were probed with primary antibodies over night at 4°C and subsequently incubated with secondary antibodies conjugated with HRP. Secondary HRP-conjugated antibodies were purchased from Dako (Heverlee, Belgium). Between probing blots were rinsed with TBST. Bands were visualized with an enhanced chemiluminescence detection system (Thermo Scientific, Amsterdam, The Netherlands). Alternatively or subsequent to probing with HRP-conjugated antibodies, secondary antibodies conjugated with IR680 or IR800 dyes were used and visualized with the Odyssey infrared detection system (Licor Westburg, Leusden, The Netherlands). For probing with Odyssey secondary antibodies we removed residual signal from HRP by washing membranes with 1% (w/v) azide in TBST for 30 minutes and subsequent probing with different primary and secondary antibodies.

### Statistical analysis

The data were analyzed using SPSS, Excel, and Graph Pad/Prism (v6.04). The zebrafish fluorescence intensity measurements were analyzed using Mann-Whitney U test. The effect of different concentrations of PAI-039 was statistically evaluated using one-way ANOVA with a Tukey post hoc test for multiple comparisons. All other analyses were performed using unpaired t-test. P-values < 0.05 were considered statistically significant (*), p-values < 0.01 were considered highly significant (**) and p-values < 0.001 were considered very highly significant (***).

## Results

### Inhibition of PAI-1 function in zebrafish leads to vascular leakage

**Fig 1:**
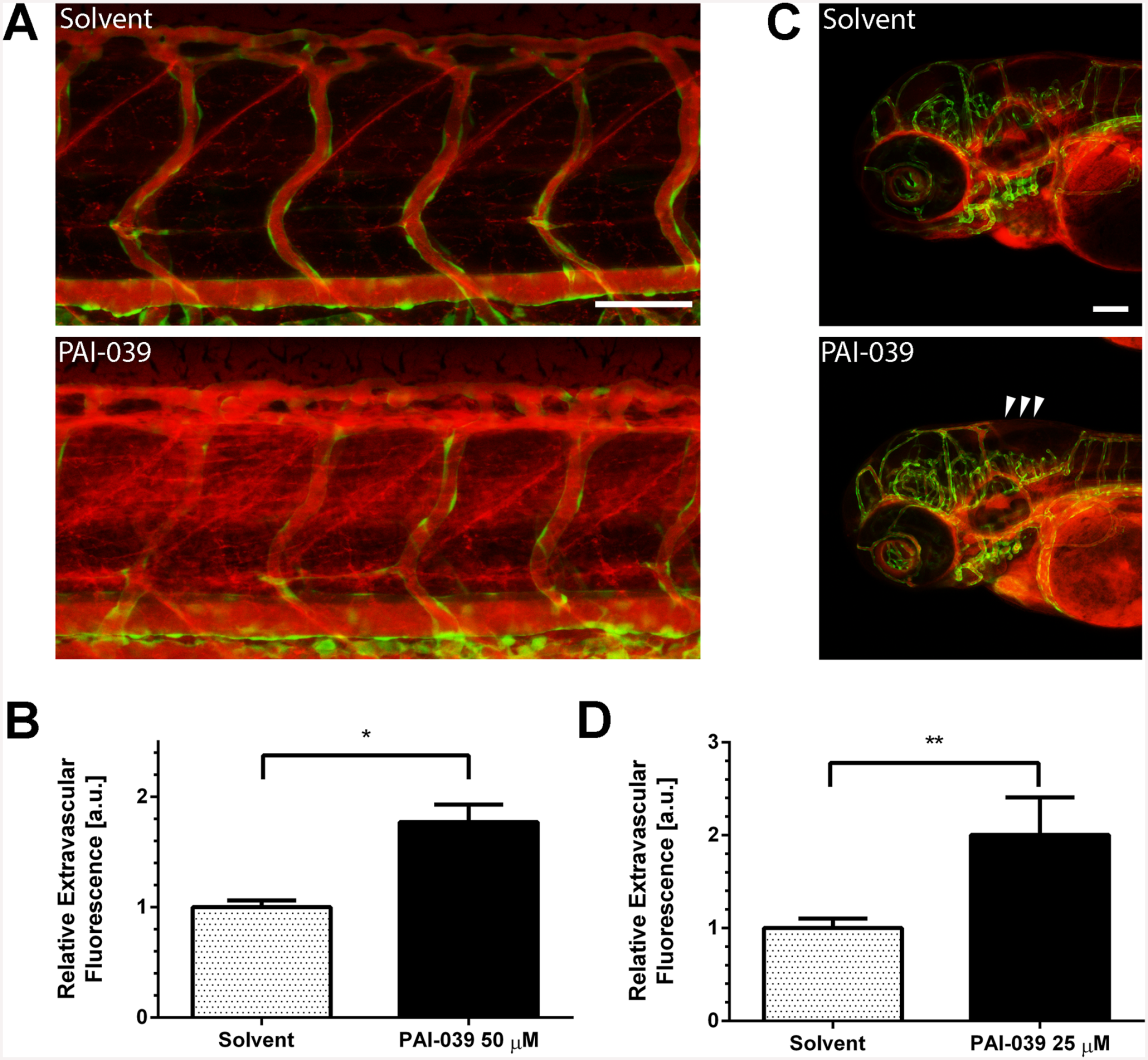
PAI-1 inhibition in zebrafish leads to vascular leakage. (A) Zebrafish embryos three-days post-fertilization (3dpf) were incubated with PAI-039 inhibitor (50 μM) or solvent (DMSO 1%) for 6 hours. Vascular leakage was visualized by injecting 2000 kDa tetramethylrhodamine(TMR)-dextran into the duct of cuvier. Intersegmental vessels are depicted in green, vascular lumen and leakage from vessels in red (scale bar 100 μm). (B) Quantification of extracellular fluorescence data from at least 15 fish were analysed for PAI-039 or solvent treatments respectively (mean + SEM, * p < 0.05). (C) 3dpf zebrafish were injected with 70 kDa TMR-dextran and incubated with PAI-039 inhibitor (25 μM) or solvent (DMSO 1%) for 3–4 hours. Vascular leakage occurs in the hindbrain of zebrafish (arrow heads) (scale bar 100 μm). (D) Quantification of extracellular fluorescence data from at least 23 fish were analysed for PAI-039 or solvent treatments respectively (mean + SEM, ** p < 0.01).

To date the increased bleeding diathesis of patients without PAI-1 has been ascribed to the importance of PAI-1 in preventing fibrinolysis. PAI-1 is a serine protease and an inhibitor of uPA and tPA and thus prevents the activation of plasminogen to plasmin. Plasmin in turn degrades fibrin clots and prevents thrombosis from overshooting fibrin formation. Thus lack of PAI-1 can lead to excessive fibrinolysis and as a consequence bleeding due to lack of wound closure. Nonetheless, there are more causes to bleeding disposition. And notably, PAI-1 is directly produced and secreted by endothelial cells and we hypothesized that defects of the vessel barrier could add to the phenotype seen in patients lacking PAI-1. To investigate this hypothesis we used PAI-039 (tiplaxtinin), a small molecule inhibitor of PAI-1, which binds to PAI-1 close to the vitronectin binding site and inhibits PAI-1 protease activity [20]. PAI-1 antagonists like PAI-039 have been successfully used to prevent thrombosis in disease models [21–23]. We first tested if PAI-1 inhibition could cause a phenotype resembling the one seen in patients, particularly hemorrhage and vascular leakage. To investigate the function of PAI-1 in vivo, we examined the effects of PAI-1 inhibiton in the zebrafish (*D*. *rerio*) vasculature. Zebrafish PAI-1 shares 42.0% sequence identity with the human homologue [24,25]. To examine if PAI-1 inhibition with PAI-039 can mimick the effects of PAI-1 deficiency in patients, we injected fluorescent TMR-dextran into zebrafish embryos to visualize any leakage from blood vessels. We observed leakage of 2000 kDa TMR-dextran from intersegmental vessels of 3dpf zebrafish embryos after six hours of treatment with 50 μM PAI-039 (Fig 1A and 1B). Further, we observed increased vascular permeability of 70 kDa dextran in the hindbrain vasculature after three to four hours of treatment with PAI-039 (25 μM) compared to controls (Fig 1C and 1D). These results indicate that, similar to the observations in patients, inhibition of PAI-1 function leads to loss of blood vessel integrity in zebrafish embryos.

### Inhibition of PAI-1 function leads to decreased transendothelial resistance

**Fig 2:**
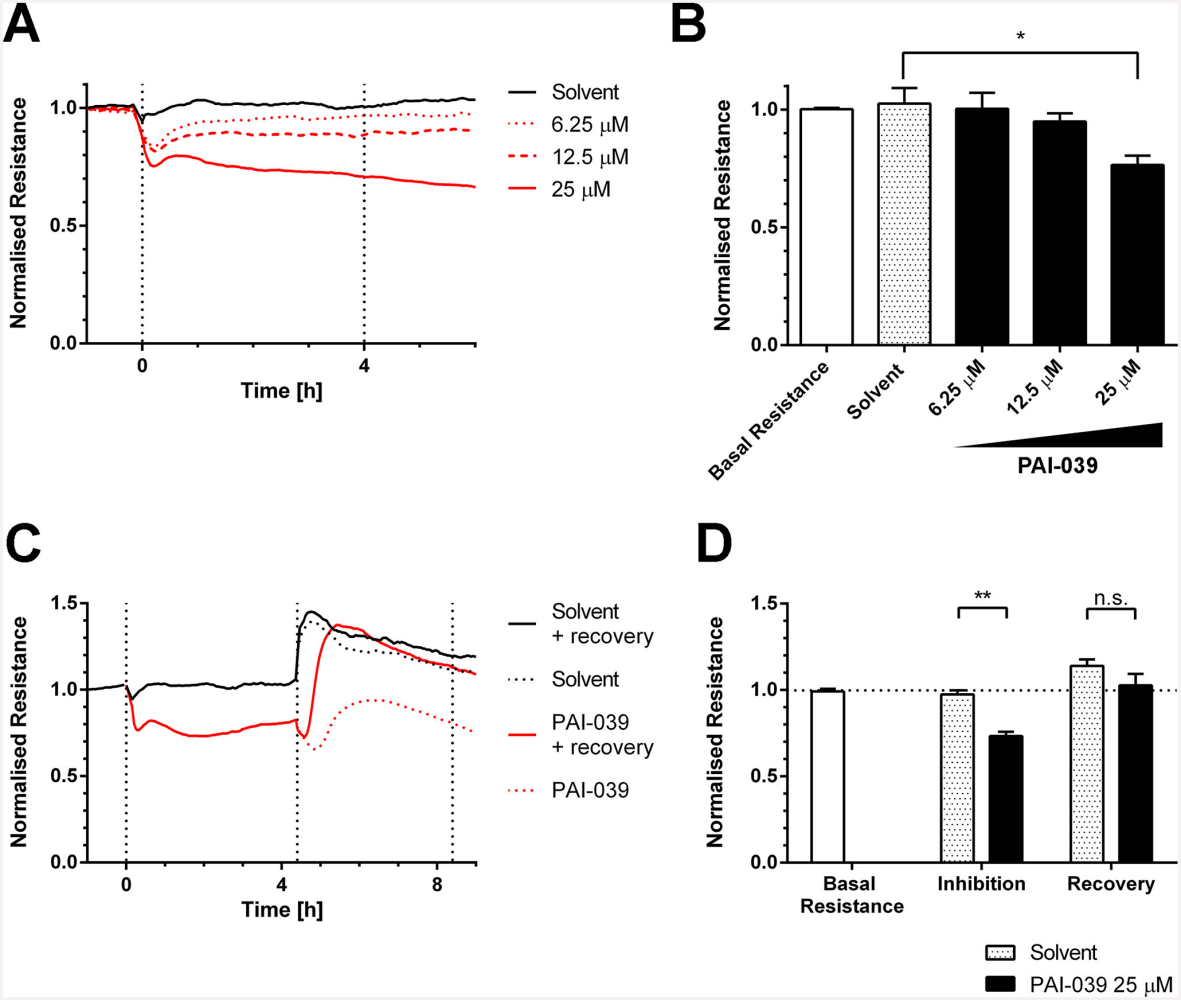
PAI-1 inhibition of HUVEC monolayers leads to loss of transendothelial resistance. (A) and (B) Transendothelial resistance (TER) was measured by electric cell-substrate impedance sensing (ECIS). HUVEC were grown to confluence in ECIS arrays and treated with either PAI-039 (6.25 μM, 12.5 μM, 25 μM) or solvent (DMSO 0.1%). Resistance values were normalized to the basal resistance one hour before addition of inhibitor. (A) is representative of one experiment (mean value of quadruplicates). (B) is representative of the normalized resistance after four hours of PAI-039 of three independent experiments, basal resistance is the normalized resistance before addition of inhibitor (mean + SEM, * p < 0.05). (C) and (D) Transendothelial resistance was measured as described in (A) and (B). After 4 hours of treatment with PAI-039 (25 μM) or solvent (DMSO 0.1%) medium was replaced with fresh medium with or without inhibitor (or solvent). TER returned to basal values within two to four hours. (C) is representative of one experiment (mean value of quadruplicates). (D) is the summary of four hour inhibition with PAI-039 (n = 10) and recovery for two hours (n = 4) (mean + SEM, ** p < 0.01).

Next we tested if PAI-1 inhibition with PAI-039 could directly affect the integrity of endothelial monolayers. The disruption of vascular integrity and increased vascular permeability are accompanied by loss of transendothelial electrical resistance. We investigated the effect of PAI-1 inhibition on HUVEC monolayers in real time with ECIS. We observed that PAI-1 inhibition with PAI-039 leads to a concentration-dependent decrease in transendothelial resistance (Fig 2A and 2B). We determined concentrations of 25 μM PAI-039 as effective in reducing transendothelial resistance of HUVEC monolayers while lower concentrations had no significant effect on transendothelial resistance. Interestingly, within less than an hour the loss in transendothelial resistance was maximal. However, when we washed out the inhibitor we observed a fast recovery of the transendothelial resistance (Fig 2C and 2D). We also tested another PAI-1 inhibitor, TM5275. TM5275 binds to different epitope of PAI-1 than PAI-039 [26]. Similar to inhibition with PAI-039 we observe a significant decrease in transendothelial resistance and a recovery after washing out of the inhibitor (S1 Fig). In line with the inhibitor data, silencing of PAI-1 using shRNA induced gaps in between the endothelial cells (S2A and S2C Fig) and reduced the basal resistance of the endothelial monolayer (S2B and S2C Fig). Note that the initial spreading of the endothelial cells was not altered in the absence of PAI-1 (S2B and S2C Fig). These experiments indicate that PAI-1 inhibition directly affects the integrity of endothelial monolayers and causes a marked decrease in endothelial resistance. The PAI-1 induced loss of barrier function, as well as its recovery are fast, indicating that PAI-1 inhibition acts very close or directly on the endothelial cell-cell junctions.

### Inhibition of PAI-1 function leads to disrupted endothelial junctions

**Fig 3:**
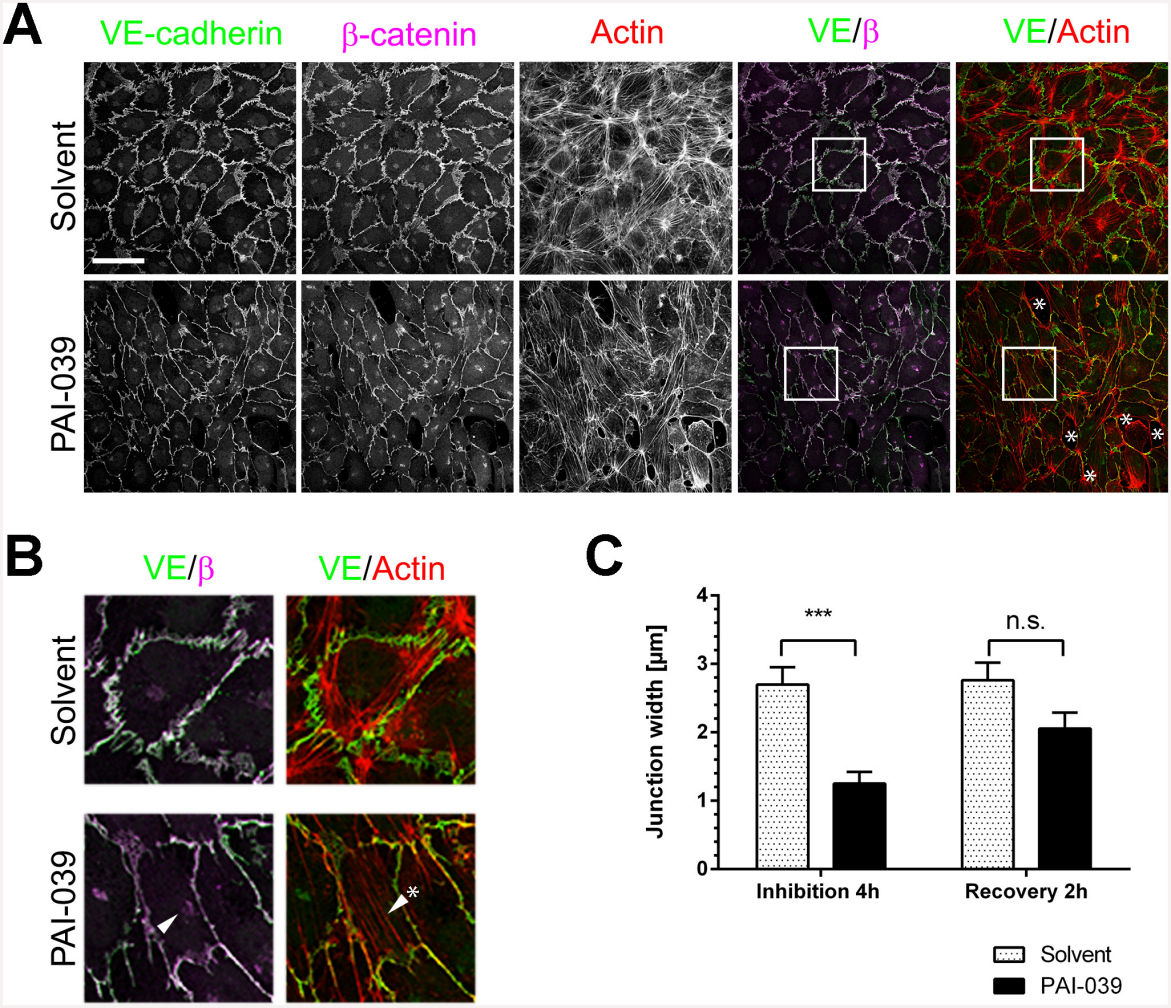
PAI-1 inhibition leads to disruption of VE-cadherin-mediated cell-cell junctions. (A) HUVEC were treated with PAI-039 (25 μM) or solvent (DMSO 0.1%) for four hours and stained for VE-cadherin (green), β-catenin (red) and actin (scale bar 50 μm). PAI-039 treatment causes disruption of junctions, gap formation (asterix) and stress fiber formation. (B) Zooms of images in (A). Arrowhead indicates accumulation of VE-cadherin in endothelial cells, arrowhead plus asterix indicates stress fibres. (C) Average junction width of cells treated with PAI-039 (25 μM) or solvent (DMSO 0.1%) for four hours (n = 4) and after junction recovery in response to washout of inhibitor (n = 2) (mean + SEM, *** p < 0.001, n.s. = non-significant).

The main regulator of endothelial integrity are adherens junctions and especially VE-cadherin which mediates homophilic binding of endothelial cells and contributes to the electrolyte barrier [27]. Blocking VE-cadherin interactions with VE-cadherin antibodies leads to a dramatic decrease in endothelial resistance [28]. To investigate if the observed decrease of endothelial transendothelial resistance was accompanied by visible disruptions to endothelial monolayers, we cultured endothelial cells to confluence and immunostained for VE-cadherin as well as α-, β- and p120 catenin and the actin cytoskeleton. We observed a marked disruption of endothelial adherens junctions and gap formation between cells after PAI-1 inhibition (Fig 3A). In controls the adherens junctions of confluent HUVEC form wide zones of overlap between endothelial cells where VE-cadherin connects adjacent cells. These junctional networks of cell-cell adhesion have been described previously as reticular junctions [29]. After PAI-1 inhibition the reticular junctions are replaced by thinner junctions (Fig 3A and 3B). After PAI-1 inhibition with PAI-039 the average junction width decreased from 2.7 μm to 1.25 μm. Within two hours of removing the inhibitor the junction width recovered (Fig 3C). We further observed accumulation of VE-cadherin around the nucleus (Fig 3B arrowhead). PAI-1 inhibition did not affect the colocalisation of VE-cadherin with α-catenin, β-catenin and p120 catenin (Fig 3A and S3A and S3B Fig). Finally, we detected stress fibers in cells treated with PAI-039 (Fig 3B, arrowhead + asterisk, S3C Fig). Stress fibers can cause gap formation by mediating contractility [30]. They are formed downstream of RhoA and Rho kinase activity [31]. We inhibited Rho kinase activation with the pharmacological inhibitor Y-27632 while at the same time inhibiting PAI-1 activity with PAI-039 and measured transendothelial resistance with ECIS. However Rho kinase inhibition with Y-27632 did not prevent the loss of transendothelial resistance (S3D Fig). In summary these results show that PAI-1 inhibition directly affects endothelial cell morphology and function by disrupting VE-cadherin-based junctions.

### PAI-1 inhibition affects VE-cadherin trafficking

**Fig 4:**
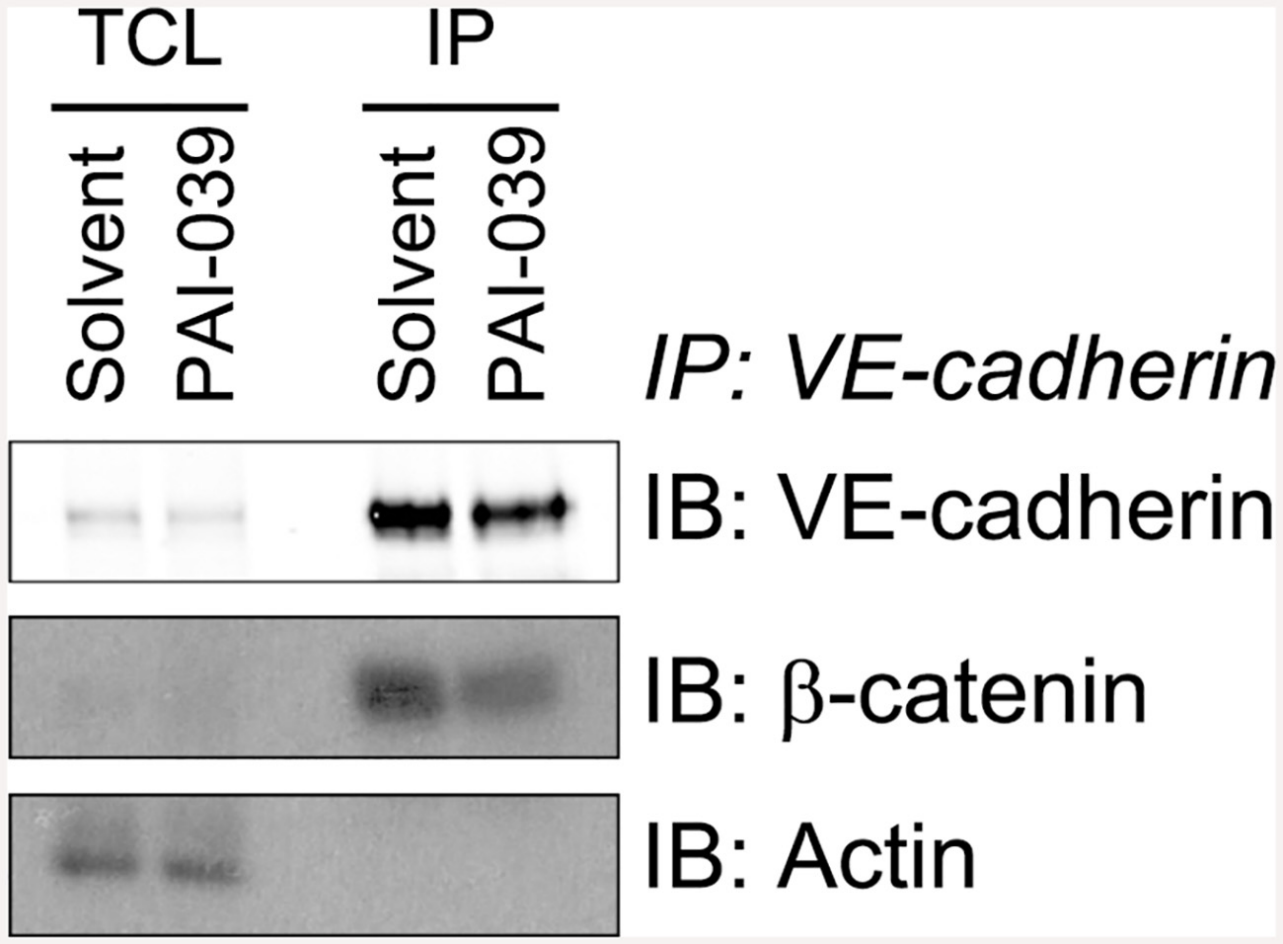
Immuno-coprecipitation of VE-cadherin from HUVEC cells after PAI-1 inhibition shows no effect on interaction of VE-cadherin with β-catenin. HUVEC were treated with PAI-039 (25 μM) or solvent (DMSO 0.1%) for four hours and total cell lysates (TCL) were subjected to immuno-coprecipitation (IP) of VE-cadherin. After SDS-PAGE proteins were blotted onto nitrocellulose membranes and the top half (> 70 kDa) first probed for β-catenin (ECL) and then for VE-cadherin (Odyssey). The bottom half (< 70 kDa) was probed for β-actin. Short and long exposures are depicted here.

**Fig 5:**
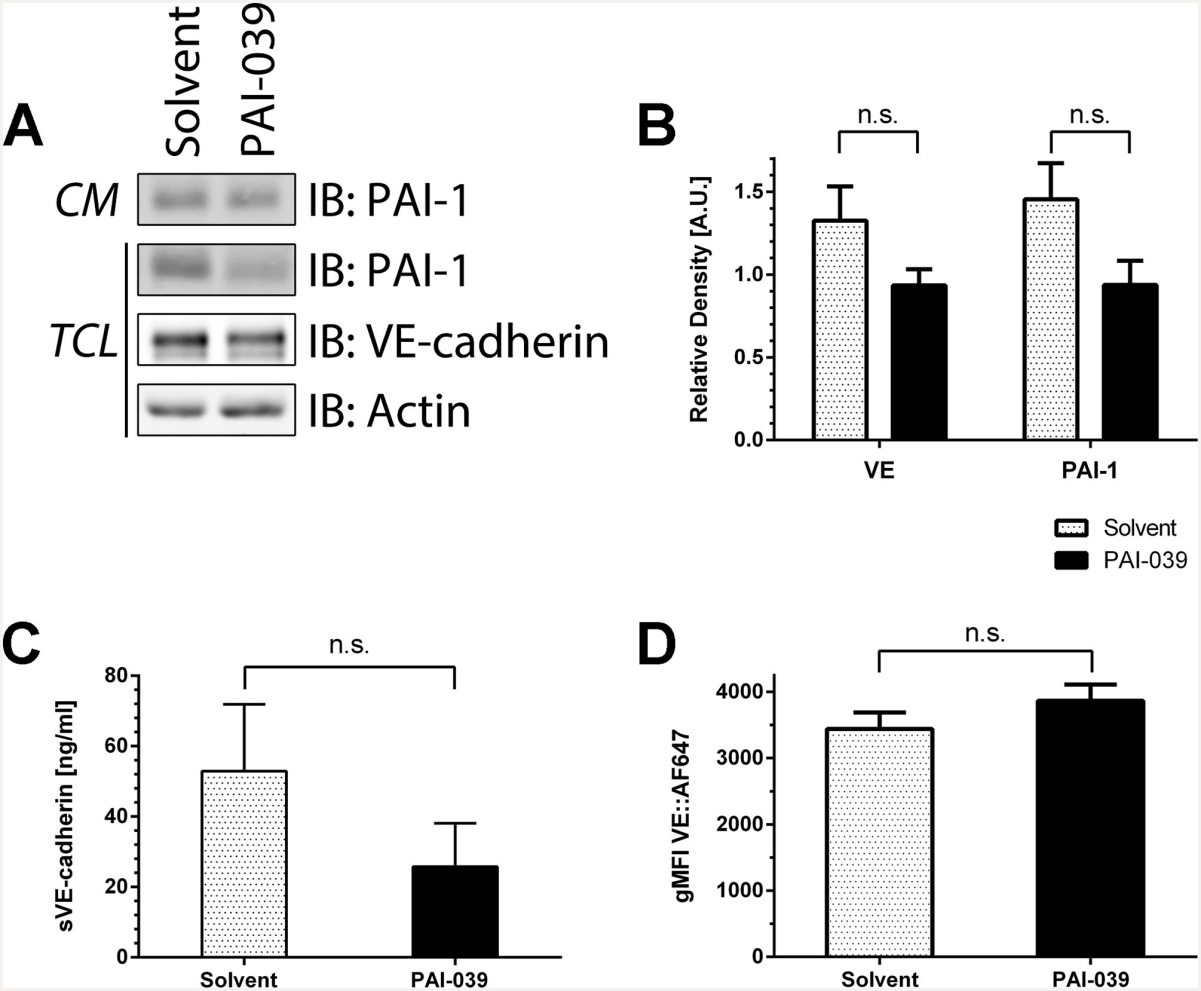
PAI-1 inhibition reduces cellular PAI-1 and VE-cadherin levels while surface levels remain unaltered. (A) HUVEC were treated with PAI-039 (25 μM) or solvent (DMSO 0.1%) for four hours and total cell lysates (TCL) and conditioned media (CM) were collected. VE-cadherin and PAI-1 levels in total cell lysates are reduced whereas secreted PAI-1 is unchanged. (B) Densitometric quantification of Western blots showed reduced (non–significant) VE-cadherin and PAI-1 after inhibitor treatment. VE-cadherin and PAI-1 levels were normalized to actin and relative density is displayed (n = 10, mean + SEM, n.s. = non-significant). (C) Levels of soluble VE-cadherin (sVE-cadherin) in conditioned media of HUVEC treated with PAI-039 or solvent (as described at (A)) were determined by ELISA. Levels of sVE-cadherin shed into conditioned media were reduced after PAI-039 treatment (n = 4, mean + SEM, n.s. = non-significant). (D) FACS analysis of HUVEC treated as described before. PAI-039 treatment had no effect on VE-cadherin surface levels (n = 3, geometric mean fluorescence intensity + SEM, n.s. = non-significant).

With immunofluorescence we observed that the colocalisation of VE-cadherin with the β-catenin, α-catenin and p120 catenin after inhibiting PAI-1 is unaltered (S4A–S4C Fig). To corroborate this finding we performed co-immunoprecipitations for VE-cadherin. We observed that VE-cadherin levels are reduced in total cell lysates and co-immunoprecipitations after PAI-039 treatment. Along with this, co-immunoprecipitation of β-catenin, α-catenin and p120 catenin are also less efficient (Fig 4, S5A and S5B Fig). Further to the reduction of VE-cadherin in total cell lysates we found a reduction of PAI-1 in total cell lysates but not in conditioned media (Fig 5A and 5B).

Since PAI-1 is a serine protease inhibitor we explored the possibility that inhibition of PAI-1 could lead to uncontrolled proteolysis of endothelial surface proteins including VE-cadherin by endothelial serine proteases [32]. VE-cadherin can be a target for proteolytic cleavage by serine proteases [33] and the extracellular domain of VE-cadherin (“soluble” VE-cadherin, sVE-cadherin) can be found in patients with inflammatory diseases like rheumatoid arthritis [34]. Therefore, we next investigated if PAI-1 inhibition leads to the release of sVE-cadherin. For this purpose we collected conditioned media from endothelial cells treated with PAI-039 and performed an ELISA specific for sVE-cadherin. To our surprise we measured less sVE-cadherin, indicating reduced shedding from cells treated with the PAI-1 inhibitor (Fig 5C). In addition we measured surface VE-cadherin expression on endothelial cells by FACS. Interestingly, PAI-1 inhibition with PAI-039 or TM5275 had no effect on the surface levels of VE-cadherin (Fig 5D, S6A Fig). Also the levels of PECAM-1, another endothelial cell-cell junction protein, were not altered (S6B and S6C Fig).

**Fig 6:**
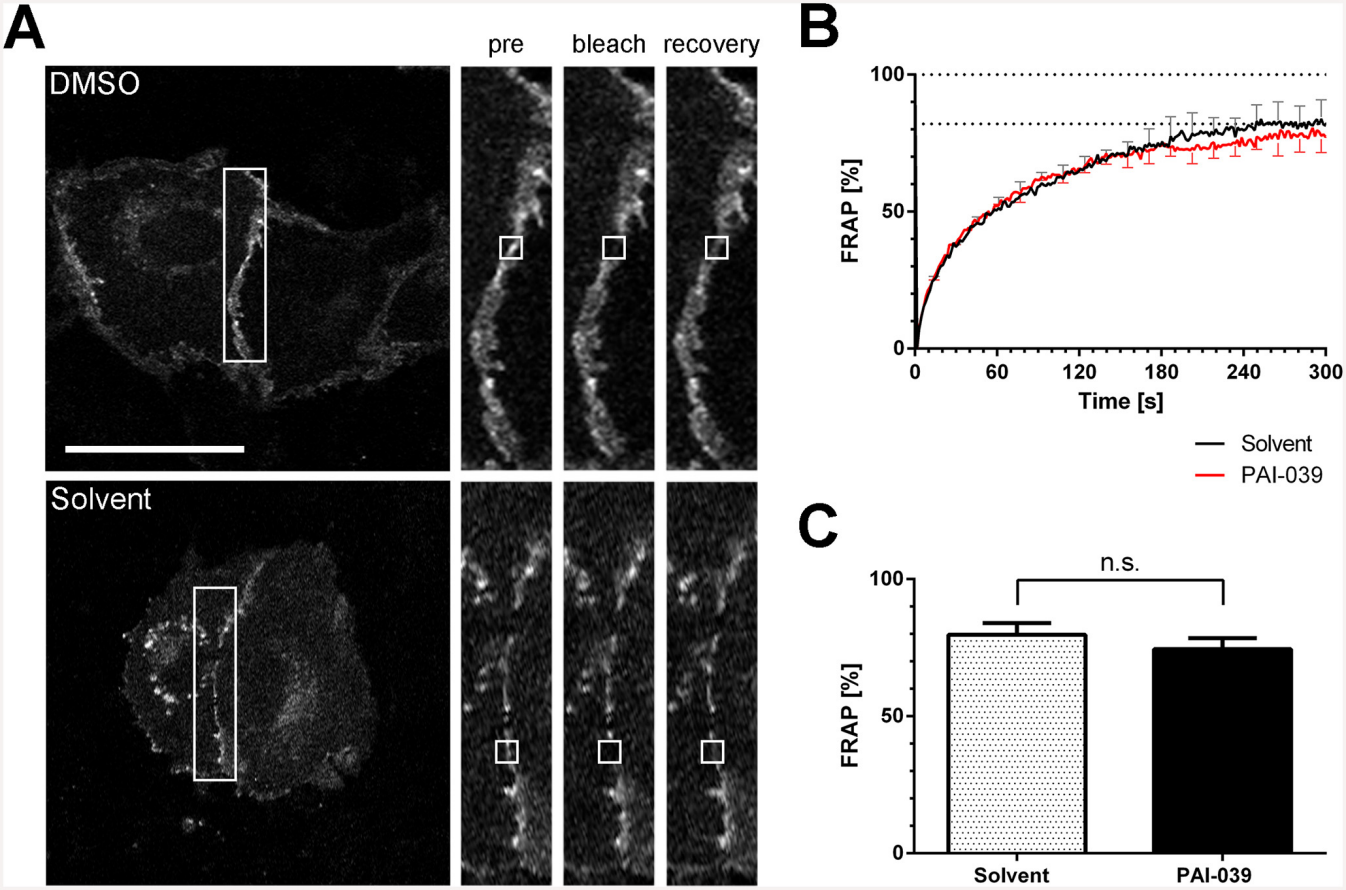
Fluorescence recovery after photobleaching (FRAP) of VE-cadherin at the plasma membrane is unaltered after PAI-1 inhibition. (A) HUVEC were transduced with lentivirus to express VE-cadherin-GFP. HUVEC were treated with PAI-039 (25 μM) or solvent (DMSO 0.1%) for two hours before FRAP imaging was performed. Fluorescence recovery was monitored for five minutes. (B) The average FRAP from five independent experiments are displayed (solvent: mean + SEM, PAI-039 mean–SEM). (C) The average FRAP from five experiments 240 seconds after bleaching is not different from solvent treated cells (mean + SEM, n.s. = non-significant).

It was shown that reduced mobility of VE-cadherin increased the resistance of endothelial monolayers [35,36]. Therefore, we next tested if PAI-1 inhibition could affect the lateral mobility of VE-cadherin at the membrane and thus disturb VE-cadherin function. We measured VE-cadherin-GFP mobility at cell-cell junctions by FRAP. For this purpose we transfected VE-cadherin-GFP into endothelial cells and inhibited PAI-1 activity with PAI-039 for two hours before FRAP measurements were made. Within four minutes VE-cadherin fluorescence recovered for both solvent- and PAI-039-treated HUVEC. There were no significant differences in the fluorescence recovery rates (Fig 6).

**Fig 7:**
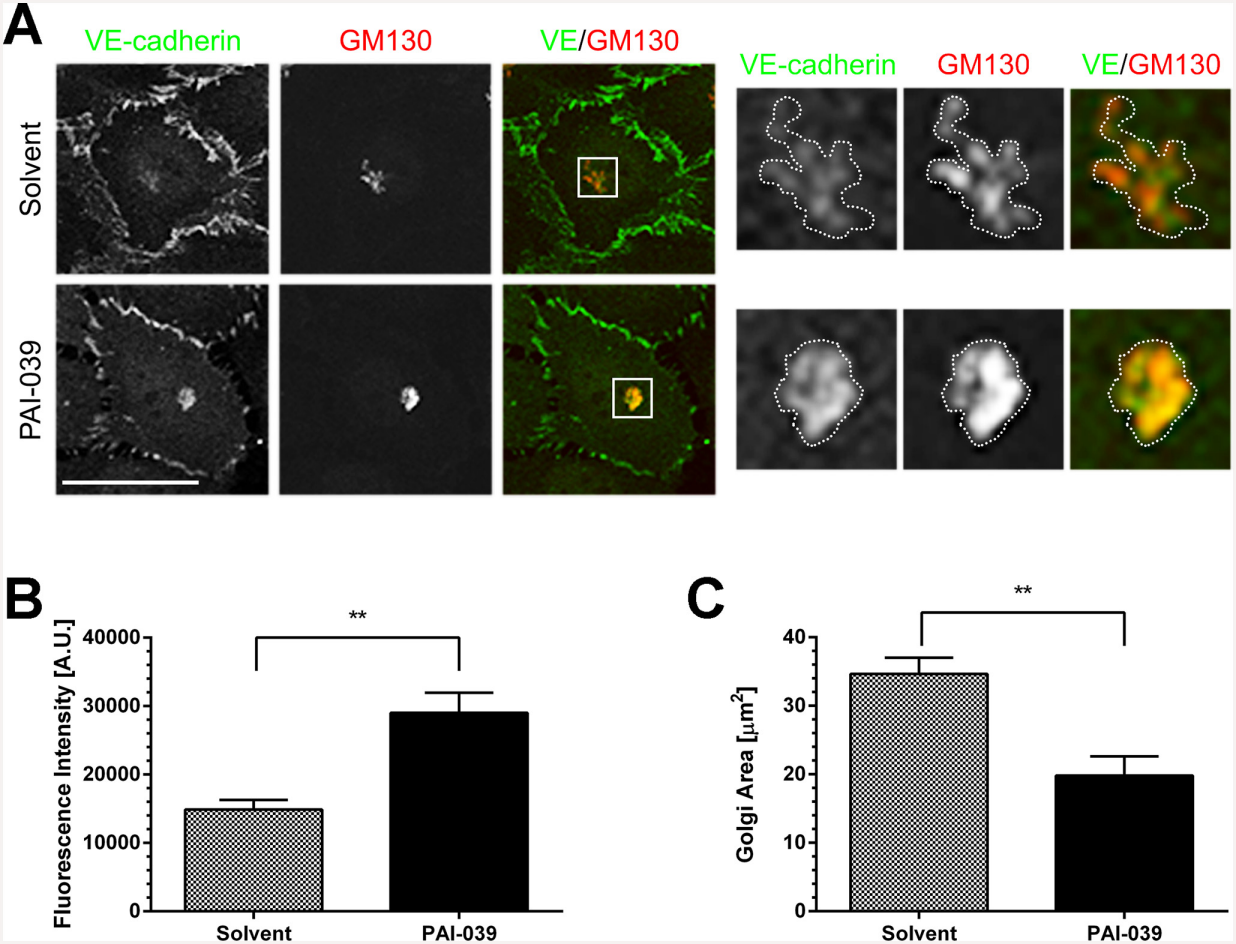
PAI-1 inhibition leads to the accumulation of VE-cadherin at the Golgi apparatus. (A) HUVEC were treated with PAI-039 (25 μM) or solvent (DMSO 0.1%) for four hours and stained for VE-cadherin (green) and GM130 (red) (scale bar 50 μm). Inset are magnified on the right side and the Golgi apparatus is outlined as for the intensity measurements. (B) PAI-039 treatment leads to the accumulation of VE-cadherin at the Golgi apparatus, (C) whereas the size of the Golgi apparatus is decreased (n = 3, mean + SEM, ** p < 0.001).

As mentioned before we noticed an accumulation of VE-cadherin in an intracellular compartment when PAI-039 was used to inhibit PAI-1 function (Fig 3B, arrowhead). Immunofluorescence co-stainings with a Golgi marker showed that VE-cadherin and β-catenin accumulation was localized at the Golgi apparatus (Fig 7A). To quantify these effects we measured the size of the Golgi apparatus and the fluorescence intensity for VE-cadherin in that compartment (Fig 7B and 7C). Interestingly, the size and fluorescence intensity at the Golgi are inversely related, with increased VE-cadherin fluorescence intensity after PAI-039 inhibition and a decrease in the size of the Golgi. Altogether the data presented here show that PAI-1 inhibition with PAI-039 leads to a reduction of the endothelial monolayer integrity by reducing total VE-cadherin levels while its surface levels and mobility remain unaltered.

## Discussion

We here present data that show a role for PAI-1 in the maintenance of vascular integrity. Our results are in agreement with the phenotype of patients that lack PAI-1 activity [15]. While PAI-1 effects on uPA and tPA are undoubtedly involved in causing the bleeding diathesis in patients with a quantitative or qualitative PAI-1 deficiency, our data suggest a more direct role of PAI-1 for maintaining the integrity of blood vessels.

The integrity of blood vessels greatly depends on the stability of cell-cell contacts between endothelial cells [37,38]. Endothelial adherens junctions and especially VE-cadherin are crucial for endothelial integrity and permeability [1]. Whereas a null mutation in VE-cadherin causes severe defects in junctional morphology and vascular morphogenesis, a partial knock-down of VE-cadherin has been shown to increased vascular permeability [39]. In addition, also merely blocking VE-cadherin with antibodies is sufficient to disrupt vascular integrity, increase permeability and cause hemorrhaging in mice [40]. Our experiments show that the inhibition of PAI-1 function with small molecule inhibitors of PAI-1 leads to disrupted endothelial junctions, which is accompanied by a marked decrease of endothelial resistance. To our knowledge this is the first time that PAI-1 function was shown to have a direct effect on endothelial junction integrity.

Quiescent endothelia are characterized by overlapping networks of reticular VE-cadherin-based adherens junctions [29]. The permeability of these types of endothelial junctions is low. We noticed that in the absence of PAI-1 activity, VE-cadherin distribution at cell-cell junctions was altered. The reticular network of VE-cadherin-based junctions was replaced by non-reticular and more “jagged” junctions. These types junctions have been described as junctions undergoing remodeling and correlate with an increase in endothelial permeability [28–30]. VE-cadherin on endothelial cells is associated with intracellular molecules like p120 catenin, β-catenin, α-catenin and actin. Particularly p120 catenin has been described as a factor in keeping VE-cadherin at the membrane by masking an endocytic signal [2,3]. However, the interaction of VE-cadherin with its intracellular binding partners was unchanged after PAI-1 inhibition.

Only few studies imply a possible connection between PAI-1 and VE-cadherin. VE-cadherin is involved in the regulation of PAI-1 transcription in endothelial cells and VE-cadherin deficient endothelial cells express less PAI-1 [41,42]. Interestingly, the inverse is also true: we show that the inhibition of PAI-1 function leads to decreased VE-cadherin levels in endothelial cells, albeit the differences we observed did not reach statistical significance. The loss of VE-cadherin expression and the disruption of the endothelial architecture occur concomitantly with the drop of endothelial resistance. It is interesting to note that both the disruption (within less than an hour) and the recovery (within two hours) of endothelial junctions occur rapidly after addition or removal, respectively, of PAI-1 inhibitors pointing at a fast regulatory mechanism. One possible mechanism for fast regulation of proteins expressed on the cell surface is ectodomain shedding by proteolytic cleavage. The surface levels of VE-cadherin can be regulated in this way [43,44]. To our surprise we did not detect increased ectodomain shedding. On the contrary, we saw less shedding while the overall surface expression of VE-cadherin remained unaltered (Fig 5). Together with our other results this suggests that the trafficking of VE-cadherin may be impaired when PAI-1 activity is inhibited.

The internalization of VE-cadherin in response to VEGF has been associated with increased permeability of endothelial monolayers [45]. Upon closer examination we observed an accumulation of VE-cadherin at the Golgi apparatus (Fig 7) which suggests that the transport of newly synthesized VE-cadherin from the Golgi to the plasma membrane may be inhibited. Interestingly, PAI-1 can be found in the Golgi of endothelial cells stimulated with LPS [46]. In our experiments we could not visualize PAI-1 by immunofluorescence, possibly due to low intracellular concentrations of PAI-1, however it would be interesting to know if PAI-1 and VE-cadherin colocalise in the Golgi apparatus. Our data suggests that PAI-1 inhibition leads to less VE-cadherin production in cells and affects the trafficking of VE-cadherin to and conceivably also from the plasma membrane.

## Concluding Remarks

PAI-1 is an interesting target for therapeutically affecting the uPA and tPA pathways that lead to plasmin activation and fibrinolysis. The use of PAI-1 antagonists, like PAI-039 and TM5275, has been praised as a promising risk-free treatment for the prevention and treatment of thrombotic disease. We here provide data that PAI-1 inhibition may not be without side-effects. In addition to the previously reported effects of PAI-1 deficiency on fibrinolysis, our data show that PAI-1 inhibition can disrupt vascular integrity. We and others have cautioned that the goal of therapeutic PAI-1 inhibition ought to be restricted to bringing PAI-1 activity levels down to physiological values [47]. This requires both careful monitoring of PAI-1 activity in plasma and a vigilant eye on vascular integrity.

## Supporting Information

S1 Fig PAI-1 inhibition of HUVEC monolayers with TM5275 leads to loss of transendothelial resistance. (A) and (B) Transendothelial electrical resistance (TER) was measured by electric cell-substrate impedance sensing (ECIS). HUVEC were grown to confluence in ECIS arrays and treated with either TM5275 (50 μM, 100 μM, 200 μM) or solvent (DMSO 0.2%). Resistance values were normalized to the basal resistance one hour before addition of inhibitor. (A) is representative of one experiment (mean value of quadruplicates). (B) is representative of normalized resistance after four hours of PAI-1 inhibition with TM5275 from the same experiment, basal resistance is the normalized resistance just before addition of inhibitor. (C) and (D) TER was measured as described in (A) and (B). After 4 hours of treatment with TM5275 (100 μM) or solvent (DMSO 0.2%) the old medium was replaced with fresh medium with or without inhibitor (or solvent). The transendothelial resistance returned to basal values within two to four hours. (C) is representative of one experiment (mean value of quadruplicates). (D) is the summary of four hour inhibition with TM5275 (n = 3) and recovery for two hours (n = 2) (mean + SEM, * p < 0.05, n.s. = non-significant).
(TIF)

S2 Fig PAI-1 depletion results in decreased electrical resistance and monolayer gaps. (A) HUVECs were grown to confluency and treated with shRNA as indicated. HUVECs depleted for PAI-1 showed gap formation. Merge shows VE-cadherin in red and F-actin in green. Bar, 10 μm. (B) Transendothelial electrical resistance (TER) was measured by electric cell-substrate impedance sensing (ECIS). HUVEC pretreated with shPAI-1 or shCTRL were plated in ECIS arrays and monitored for resistance. No change in the initial spreading of the cells was detected; however, when forming a stable monolayer, a reduced resistance was measured for HUVECs that were depleted for PAI-1. (C) Quantification of resistance of HUVECs after 20 hours of plating. Data are mean ± s.e.m. Experiment is carried out three times independently form each other and per experiment in duplicate. (D) Western blot analysis shows efficient depletion of PAI-1. Actin is shown as loading control.
(TIF)

S3 Fig PAI-1 inhibition leads to disruption of VE-cadherin-mediated cell junctions. (A) and (B) HUVEC were treated with PAI-039 (25 μM) or solvent (DMSO 0.1%) for four hours and stained for VE-cadherin (green), actin and (A) p120 catenin or (B) α-catenin (red) (scale bar 50 μm). (C) Illustration of junction width measurements. Five junctional measurements per cells were taken as shown in this picture. (D) TER was measured by ECIS. HUVEC were grown to confluence in ECIS arrays and treated with either PAI-039 (25 μM) or Control (DMSO 0.1%) and Y-27632 (10 μM) as indicated. Resistance values were normalized to the basal resistance one hour before addition of inhibitors. Treatment with Y-27632 did not prevent decrease in TER after PAI-039 treatment. Graph is representative of one experiment (mean value of quadruplicates + SD).
(TIF)

S4 Fig PAI-1 inhibition of HUVEC monolayers with TM5275 leads to loss of transendothelial resistance. HUVEC were treated with TM5275 (50 μM) or solvent (DMSO 0.1%) for four hours and stained for VE-cadherin (green), actin and (A) β-catenin, (B) p120 catenin, or (C) α-catenin (red) (scale bar 50 μm). Junctions were disrupted and stress fibres were formed. For control see main text and Fig 3.
(TIF)

S5 Fig PAI-1 inhibition with PAI-039 or TM5275 does not alter VE-cadherin and PECAM-1 expression on HUVEC. (A), (B), (C) FACS analysis of HUVEC treated with TM5275 (100 μM, control: DMSO 0.2%) or PAI-039 (25 μM, control: DMSO 0.1%) as indicated. PAI-1 inhibition did not affect VE-cadherin expression on HUVEC (n = 3, geometric mean fluorescence intensity + SEM, n.s. = non-significant). (D) Densitometric quantification of PAI-1 in conditioned media of HUVEC treated with PAI-039 as described in Fig 5B (n = 3, mean + SEM, n.s. = non-significant).
(TIF)

S6 Fig Immuno-coprecipitation of VE-cadherin from HUVEC cells after PAI-1 inhibition shows no effect on interaction of VE-cadherin with α-catenin or p120 catenin. HUVEC were treated with PAI-039 (25 μM) or solvent (DMSO 0.1%) for four hours and total cell lysates (TCL) were subjected to immuno-coprecipitation (IP) of VE-cadherin. After SDS-PAGE proteins were blotted onto nitrocellulose membranes and the top half (> 70 kDa) first probed for (A) α-catenin or (B) p120 catenin (ECL) and then for VE-cadherin (Odyssey). The bottom half (< 70 kDa) was probed for β-actin. Short and long exposures are depicted here.
(TIF)

## References

[1] Dejana E, Orsenigo F, Lampugnani MG. The role of adherens junctions and VE-cadherin in the control of vascular permeability. JCell Sci 2008;121:2115–22. 18565824 10.1242/jcs.017897

[2] Xiao K, Allison DF, Buckley KM, Kottke MD, Vincent PA, Faundez V, et al Cellular levels of p120 catenin function as a set point for cadherin expression levels in microvascular endothelial cells. JCell Biol 2003;163:535–45. 14610056 10.1083/jcb.200306001 PMC2173638

[3] Nanes BA, Chiasson-Mackenzie C, Lowery AM, Ishiyama N, Faundez V, Ikura M, et al p120-catenin binding masks an endocytic signal conserved in classical cadherins. JCell Biol 2012;199:365–80. 23071156 10.1083/jcb.201205029 PMC3471230

[4] Ukropec JA, Hollinger MK, Woolkalis MJ. Regulation of VE-cadherin linkage to the cytoskeleton in endothelial cells exposed to fluid shear stress. ExpCell Res 2002;273:240–7. 10.1006/excr.2001.5453 11822879

[5] Lampugnani MG, Corada M, Caveda L, Breviario F, Ayalon O, Geiger B, et al The molecular organization of endothelial cell to cell junctions: differential association of plakoglobin, beta-catenin, and alpha-catenin with vascular endothelial cadherin (VE-cadherin). JCell Biol 1995;129:203–17. 7698986 10.1083/jcb.129.1.203 PMC2120375

[6] Hofmann S, Grasberger H, Jung P, Bidlingmaier M, Vlotides J, Janssen OE, et al The tumour necrosis factor-alpha induced vascular permeability is associated with a reduction of VE-cadherin expression. Eur J Med Res 2002;7:171–6. 12010652

[7] Nwariaku FE, Chang J, Zhu X, Liu Z, Duffy SL, Halaihel NH, et al The role of p38 map kinase in tumor necrosis factor-induced redistribution of vascular endothelial cadherin and increased endothelial permeability. Shock 2002;18:82–5. 12095140 10.1097/00024382-200207000-00015

[8] Dellas C, Loskutoff DJ. Historical analysis of PAI-1 from its discovery to its potential role in cell motility and disease. ThrombHaemost 2005;93:631–40. 10.1160/TH05-01-0033 15841306

[9] Chen S-C, Henry DO, Reczek PR, Wong MKK. Plasminogen activator inhibitor-1 inhibits prostate tumor growth through endothelial apoptosis. Mol Cancer Ther 2008;7:1227–36. 10.1158/1535-7163.MCT-08-0051 18483310

[10] Ploplis VA, Balsara R, Sandoval-Cooper MJ, Yin ZJ, Batten J, Modi N, et al Enhanced in vitro proliferation of aortic endothelial cells from plasminogen activator inhibitor-1-deficient mice. J Biol Chem 2004;279:6143–51. 10.1074/jbc.M307297200 14625301

[11] Isogai C, Laug WE, Shimada H, Declerck PJ, Stins MF, Durden DL, et al Plasminogen activator inhibitor-1 promotes angiogenesis by stimulating endothelial cell migration toward fibronectin. Cancer Res 2001;61:5587–94. 11454712

[12] Degryse B, Neels JG, Czekay R-P, Aertgeerts K, Kamikubo Y-I, Loskutoff DJ. The low density lipoprotein receptor-related protein is a motogenic receptor for plasminogen activator inhibitor-1. J Biol Chem 2004;279:22595–604. 10.1074/jbc.M313004200 15001579

[13] Soeda S, Oda M, Ochiai T, Shimeno H. Deficient release of plasminogen activator inhibitor-1 from astrocytes triggers apoptosis in neuronal cells. Brain Res Mol Brain Res 2001;91:96–103. 11457496 10.1016/s0169-328x(01)00133-4

[14] Czekay RP, Aertgeerts K, Curriden SA, Loskutoff DJ. Plasminogen activator inhibitor-1 detaches cells from extracellular matrices by inactivating integrins. J Cell Biol 2003;160:781–91. 10.1083/jcb.200208117 12615913 PMC2173358

[15] Mehta R, Shapiro AD. Plasminogen activator inhibitor type 1 deficiency. Haemophilia 2008;14:1255–60. 10.1111/j.1365-2516.2008.01834.x 19141166

[16] Jin S, Beis D, Mitchell T, Chen J, Stainier DYR. Cellular and molecular analyses of vascular tube and lumen formation in zebrafish. Development 2005;132:5199–209. 10.1242/dev.02087 16251212

[17] Westerfield M. The Zebrafish Book A Guide for the Laboratory Use of Zebrafish (Danio rerio). 4th editio. Eugene, Oregon, USA: University of Origon Press, Eugene; 2000.

[18] Isogai S, Horiguchi M, Weinstein BM. The vascular anatomy of the developing zebrafish: an atlas of embryonic and early larval development. Dev Biol 2001;230:278–301. 10.1006/dbio.2000.9995 11161578

[19] Timmerman I, Heemskerk N, Kroon J, Schaefer A, van Rijssel J, Hoogenboezem M, et al A local VE-cadherin/Trio-based signaling complex stabilizes endothelial junctions through Rac1 2015. 10.1242/jcs.168674 26116572

[20] Gorlatova NV, Cale JM, Elokdah H, Li D, Fan K, Warnock M, et al Mechanism of inactivation of plasminogen activator inhibitor-1 by a small molecule inhibitor. JBiolChem 2007;282:9288–96. 10.1074/jbc.M611642200 17276980

[21] Hennan JK, Morgan GA, Swillo RE, Antrilli TM, Mugford C, Vlasuk GP, et al Effect of tiplaxtinin (PAI-039), an orally bioavailable PAI-1 antagonist, in a rat model of thrombosis. JThrombHaemost 2008;6:1558–64. 10.1111/j.1538-7836.2008.03063.x 18624980

[22] Elokdah H, Abou-Gharbia M, Hennan JK, McFarlane G, Mugford CP, Krishnamurthy G, et al Tiplaxtinin, a novel, orally efficacious inhibitor of plasminogen activator inhibitor-1: design, synthesis, and preclinical characterization. JMedChem 2004;47:3491–4. 10.1021/jm049766q 15214776

[23] Baxi S, Crandall DL, Meier TR, Wrobleski S, Hawley A, Farris D, et al Dose-dependent thrombus resolution due to oral plaminogen activator inhibitor (PAI)-1 inhibition with tiplaxtinin in a rat stenosis model of venous thrombosis. Thromb Haemost 2008;99:749–58. 10.1160/TH07-11-0669 18392333

[24] Bager R, Johansen JS, Jensen JK, Stensballe A, Jendroszek A, Buxbom L, et al Protein conformational change delayed by steric hindrance from an N-linked glycan. J Mol Biol 2013;425:2867–77. 10.1016/j.jmb.2013.05.007 23702291

[25] Lang MR, Gihr G, Gawaz MP, Müller II. Hemostasis in Danio rerio: is the zebrafish a useful model for platelet research? J Thromb Haemost 2010;8:1159–69. 10.1111/j.1538-7836.2010.03815.x 20180901

[26] Izuhara Y, Yamaoka N, Kodama H, Dan T, Takizawa S, Hirayama N, et al A novel inhibitor of plasminogen activator inhibitor-1 provides antithrombotic benefits devoid of bleeding effect in nonhuman primates. JCerebBlood Flow Metab 2010;30:904–12. 10.1038/jcbfm.2009.272 PMC2949193 20087372

[27] Dejana E, Tournier-Lasserve E, Weinstein BM. The control of vascular integrity by endothelial cell junctions: molecular basis and pathological implications. Dev Cell 2009;16:209–21. 10.1016/j.devcel.2009.01.004 19217423

[28] Hordijk PL, Anthony E, Mul FP, Rientsma R, Oomen LC, Roos D. Vascular-endothelial-cadherin modulates endothelial monolayer permeability. JCell Sci 1999;112(Pt 1):1915–23. 10341210 10.1242/jcs.112.12.1915

[29] Fernandez-Martin L, Marcos-Ramiro B, Bigarella CL, Graupera M, Cain RJ, Reglero-Real N, et al Crosstalk Between Reticular Adherens Junctions and Platelet Endothelial Cell Adhesion Molecule-1 Regulates Endothelial Barrier Function. ArteriosclerThrombVascBiol 2012. 10.1161/ATVBAHA.112.252080 22723439

[30] Huveneers S, Oldenburg J, Spanjaard E, van der Krogt G, Grigoriev I, Akhmanova A, et al Vinculin associates with endothelial VE-cadherin junctions to control force-dependent remodeling. J Cell Biol 2012;196:641–52. 10.1083/jcb.201108120 22391038 PMC3307691

[31] Van Nieuw Amerongen GP, van Delft S, Vermeer MA, Collard JG, van Hinsbergh VW. Activation of RhoA by thrombin in endothelial hyperpermeability: role of Rho kinase and protein tyrosine kinases. Circ Res 2000;87:335–40. 10948069 10.1161/01.res.87.4.335

[32] Aimes RT, Zijlstra A, Hooper JD, Ogbourne SM, Sit M-L, Fuchs S, et al Endothelial cell serine proteases expressed during vascular morphogenesis and angiogenesis. Thromb Haemost 2003;89:561–72. 12624642

[33] Attali C, Durmort C, Vernet T, Di Guilmi AM. The interaction of Streptococcus pneumoniae with plasmin mediates transmigration across endothelial and epithelial monolayers by intercellular junction cleavage. Infect Immun 2008;76:5350–6. 10.1128/IAI.00184-08 18725422 PMC2573366

[34] Sidibe A, Mannic T, Arboleas M, Subileau M, Gulino-Debrac D, Bouillet L, et al Soluble VE-cadherin in rheumatoid arthritis patients correlates with disease activity: evidence for tumor necrosis factor alpha-induced VE-cadherin cleavage. Arthritis Rheum 2012;64:77–87. 10.1002/art.33336 21905018

[35] Noda K, Zhang J, Fukuhara S, Kunimoto S, Yoshimura M, Mochizuki N. Vascular endothelial-cadherin stabilizes at cell-cell junctions by anchoring to circumferential actin bundles through alpha- and beta-catenins in cyclic AMP-Epac-Rap1 signal-activated endothelial cells. MolBiolCell 2010;21:584–96. 10.1091/mbc.E09-07-0580 PMC2820423 20032304

[36] Kooistra MR, Corada M, Dejana E, Bos JL. Epac1 regulates integrity of endothelial cell junctions through VE-cadherin. FEBS Lett 2005;579:4966–72. 16115630 10.1016/j.febslet.2005.07.080

[37] Dejana E, Simionescu M, Wolburg H. Endothelial cell biology and pathology. Cell Tissue Res 2009;335:1–3. 10.1007/s00441-008-0697-2 19015887

[38] Trani M, Dejana E. New insights in the control of vascular permeability: vascular endothelial-cadherin and other players. Curr Opin Hematol 2015;22:267–72. 10.1097/MOH.0000000000000137 25767951

[39] Montero-Balaguer M, Swirsding K, Orsenigo F, Cotelli F, Mione M, Dejana E. Stable vascular connections and remodeling require full expression of VE-cadherin in zebrafish embryos. PLoS One 2009;4:e5772 10.1371/journal.pone.0005772 19503615 PMC2685470

[40] Corada M, Mariotti M, Thurston G, Smith K, Kunkel R, Brockhaus M, et al Vascular endothelial-cadherin is an important determinant of microvascular integrity in vivo. ProcNatlAcadSciUSA 1999;96:9815–20. 10.1073/pnas.96.17.9815 PMC22293 10449777

[41] Zanetta L, Corada M, Grazia LM, Zanetti A, Breviario F, Moons L, et al Downregulation of vascular endothelial-cadherin expression is associated with an increase in vascular tumor growth and hemorrhagic complications. ThrombHaemost 2005;93:1041–6. 10.1160/TH04-10-0680 15968386

[42] Rudini N, Felici A, Giampietro C, Lampugnani M, Corada M, Swirsding K, et al VE-cadherin is a critical endothelial regulator of TGF-beta signalling. EMBO J 2008;27:993–1004. 10.1038/emboj.2008.46 18337748 PMC2323269

[43] Herren B, Levkau B, Raines EW, Ross R. Cleavage of beta-catenin and plakoglobin and shedding of VE-cadherin during endothelial apoptosis: evidence for a role for caspases and metalloproteinases. MolBiolCell 1998;9:1589–601. 10.1091/mbc.9.6.1589 PMC25388 9614196

[44] Navaratna D, McGuire PG, Menicucci G, Das A. Proteolytic degradation of VE-cadherin alters the blood-retinal barrier in diabetes. Diabetes 2007;56:2380–7. 17536065 10.2337/db06-1694

[45] Gavard J, Gutkind JS. VEGF controls endothelial-cell permeability by promoting the beta-arrestin-dependent endocytosis of VE-cadherin. NatCell Biol 2006;8:1223–34. 10.1038/ncb1486 17060906

[46] Boncela J, Przygodzka P, Papiewska-Pajak I, Wyroba E, Osinska M, Cierniewski CS. Plasminogen activator inhibitor type 1 interacts with alpha3 subunit of proteasome and modulates its activity. JBiolChem 2011;286:6820–31. 10.1074/jbc.M110.173781 PMC3057828 21135093

[47] Vaughan DE. PAI-1 antagonists: the promise and the peril. Trans Am Clin Climatol Assoc 2011;122:312–25. 21686234 PMC3116335

