# Supplementary figures and images for: Plasminogen Activator Inhibitor-1 Controls Vascular Integrity by Regulating VE-Cadherin Trafficking
Source: PLoS One. 2015 Dec 29;10(12):e0145684. doi: 10.1371/journal.pone.0145684 (PMC4694698; doi:10.1371/journal.pone.0145684)

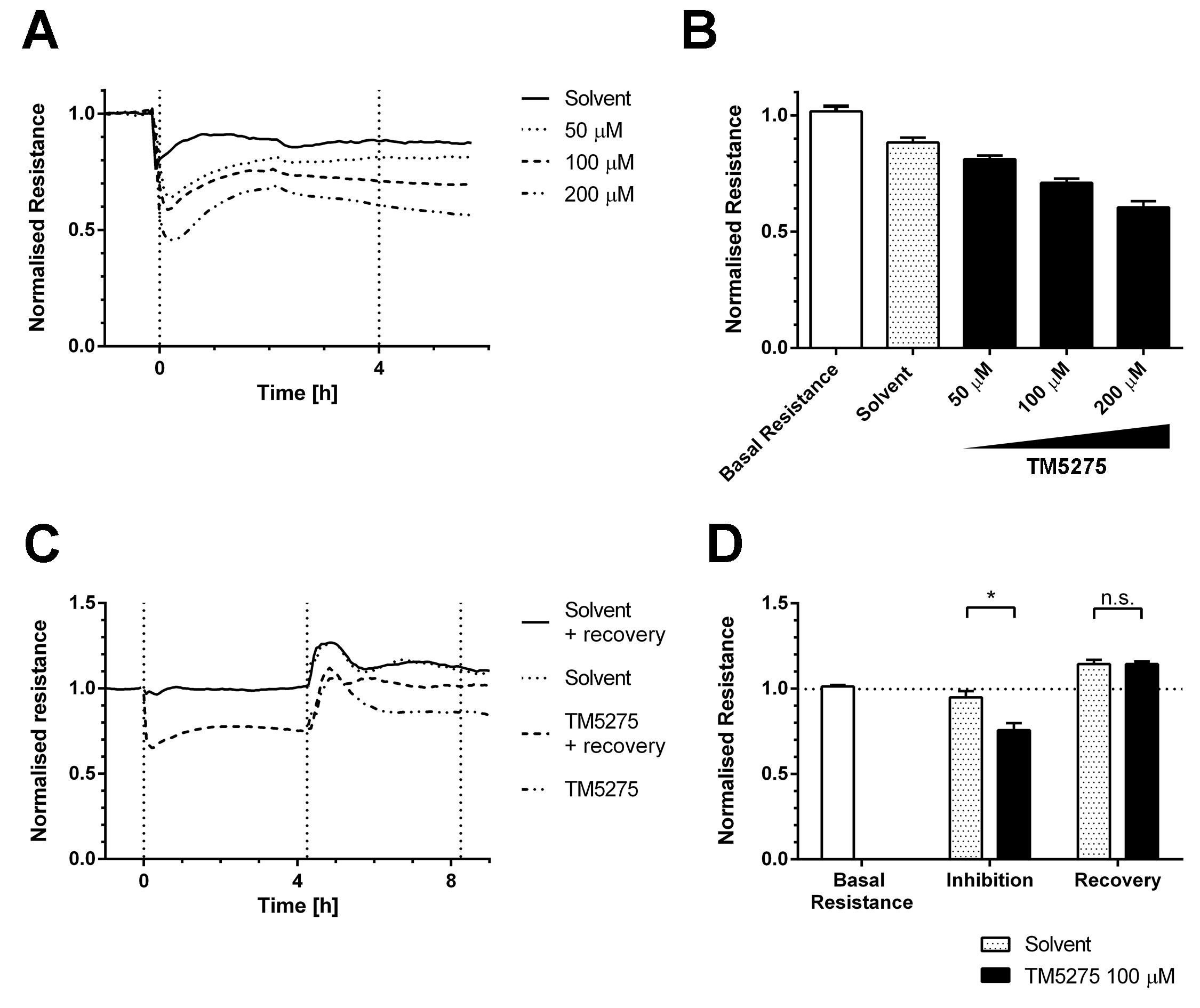

Supplement: S1 Fig — (A) and (B) Transendothelial electrical resistance (TER) was measured by electric cell-substrate impedance sensing (ECIS). HUVEC were grown to confluence in ECIS arrays and treated with either TM5275 (50 μM, 100 μM, 200 μM) or solvent (DMSO 0.2%). Resistance values were normalized to the basal resistance one hour before addition of inhibitor. (A) is representative of one experiment (mean value of quadruplicates). (B) is representative of normalized resistance after four hours of PAI-1 inhibition with TM5275 from the same experiment, basal resistance is the normalized resistance just before addition of inhibitor. (C) and (D) TER was measured as described in (A) and (B). After 4 hours of treatment with TM5275 (100 μM) or solvent (DMSO 0.2%) the old medium was replaced with fresh medium with or without inhibitor (or solvent). The transendothelial resistance returned to basal values within two to four hours. (C) is representative of one experiment (mean value of quadruplicates). (D) is the summary of four hour inhibition with TM5275 (n = 3) and recovery for two hours (n = 2) (mean + SEM, * p < 0.05, n.s. = non-significant). (TIF) [file pone.0145684.s001.tif]

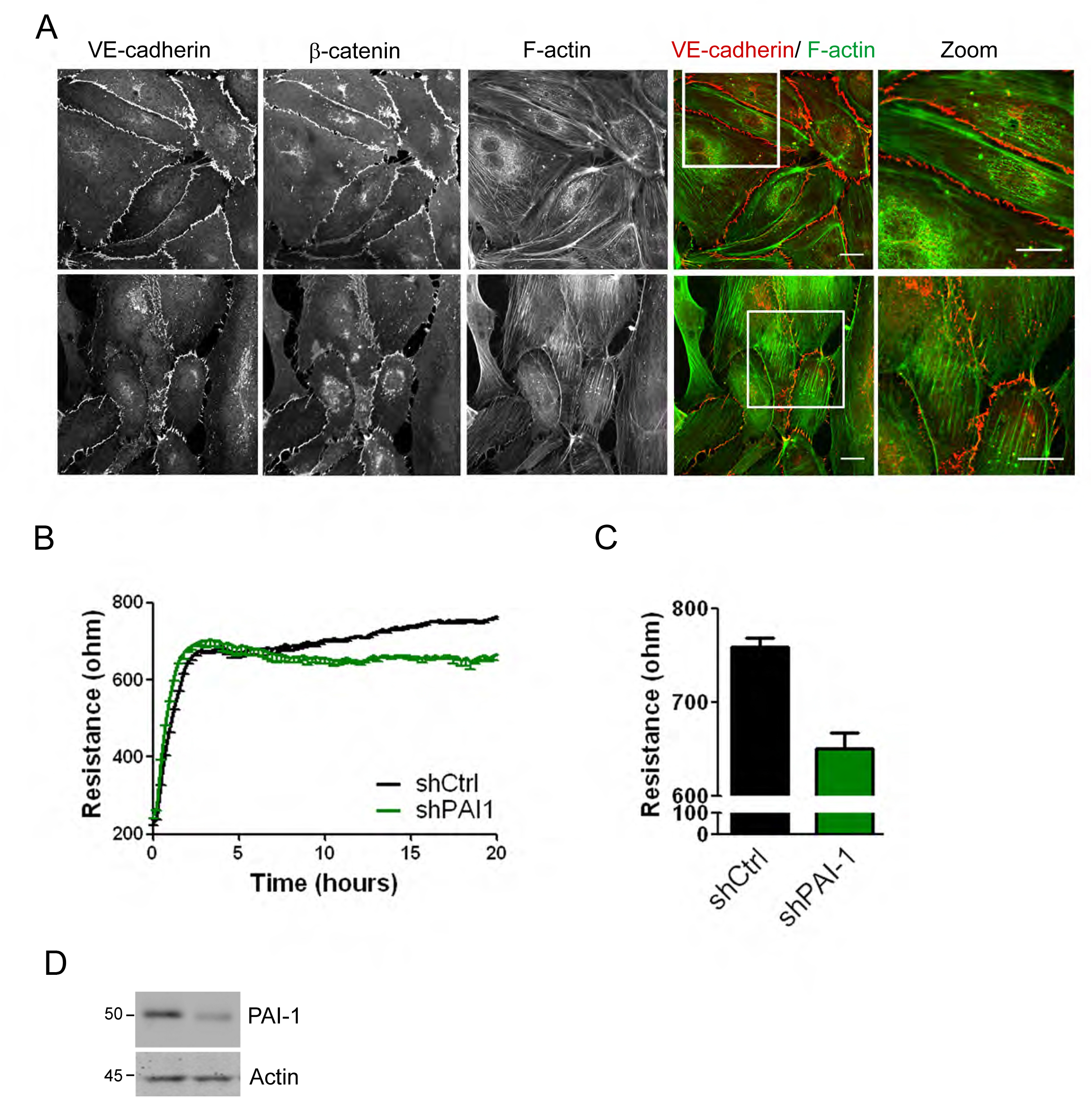

Supplement: S2 Fig — (A) HUVECs were grown to confluency and treated with shRNA as indicated. HUVECs depleted for PAI-1 showed gap formation. Merge shows VE-cadherin in red and F-actin in green. Bar, 10 μm. (B) Transendothelial electrical resistance (TER) was measured by electric cell-substrate impedance sensing (ECIS). HUVEC pretreated with shPAI-1 or shCTRL were plated in ECIS arrays and monitored for resistance. No change in the initial spreading of the cells was detected; however, when forming a stable monolayer, a reduced resistance was measured for HUVECs that were depleted for PAI-1. (C) Quantification of resistance of HUVECs after 20 hours of plating. Data are mean ± s.e.m. Experiment is carried out three times independently form each other and per experiment in duplicate. (D) Western blot analysis shows efficient depletion of PAI-1. Actin is shown as loading control. (TIF) [file pone.0145684.s002.tif]

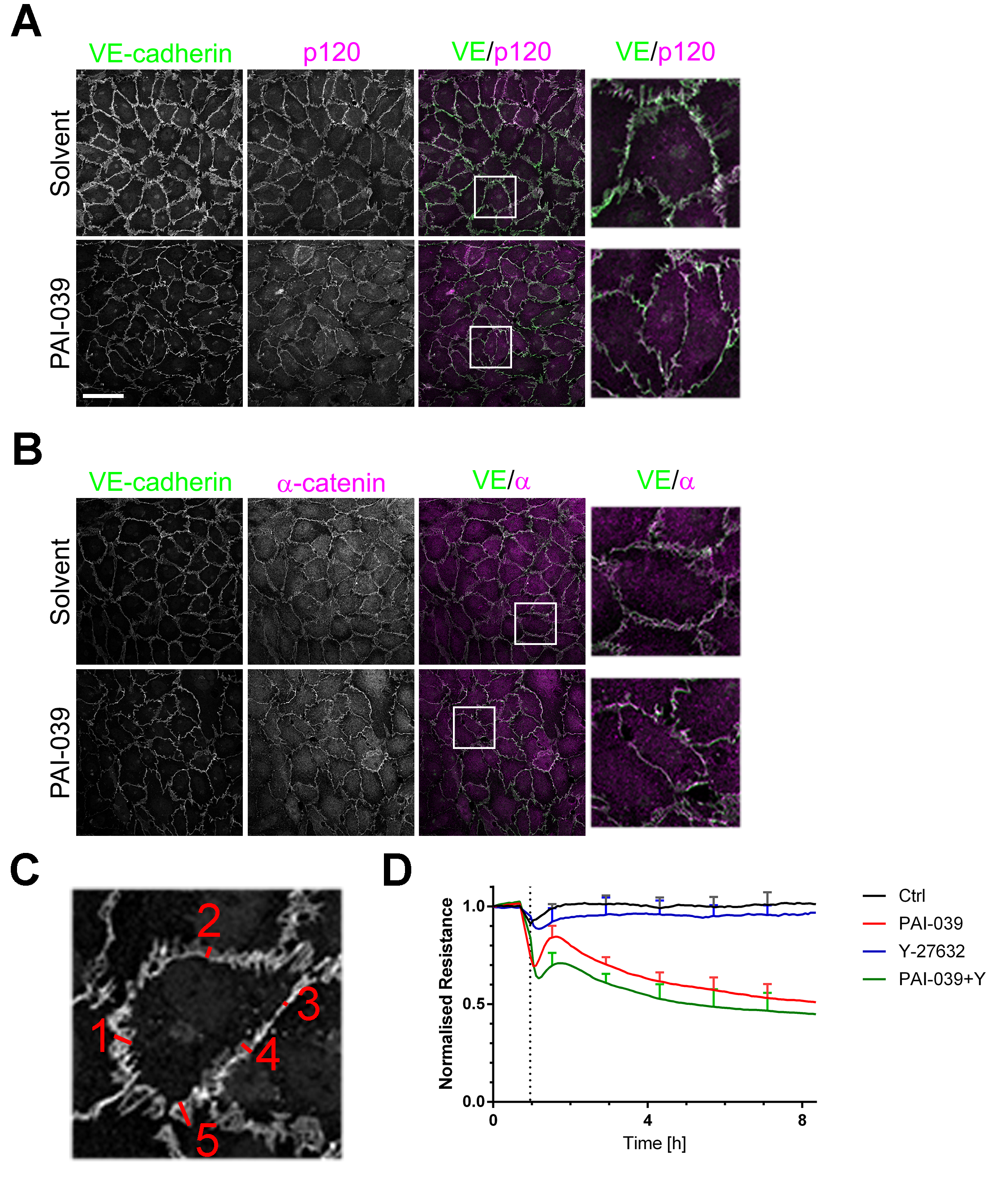

Supplement: S3 Fig — (A) and (B) HUVEC were treated with PAI-039 (25 μM) or solvent (DMSO 0.1%) for four hours and stained for VE-cadherin (green), actin and (A) p120 catenin or (B) α-catenin (red) (scale bar 50 μm). (C) Illustration of junction width measurements. Five junctional measurements per cells were taken as shown in this picture. (D) TER was measured by ECIS. HUVEC were grown to confluence in ECIS arrays and treated with either PAI-039 (25 μM) or Control (DMSO 0.1%) and Y-27632 (10 μM) as indicated. Resistance values were normalized to the basal resistance one hour before addition of inhibitors. Treatment with Y-27632 did not prevent decrease in TER after PAI-039 treatment. Graph is representative of one experiment (mean value of quadruplicates + SD). (TIF) [file pone.0145684.s003.tif]

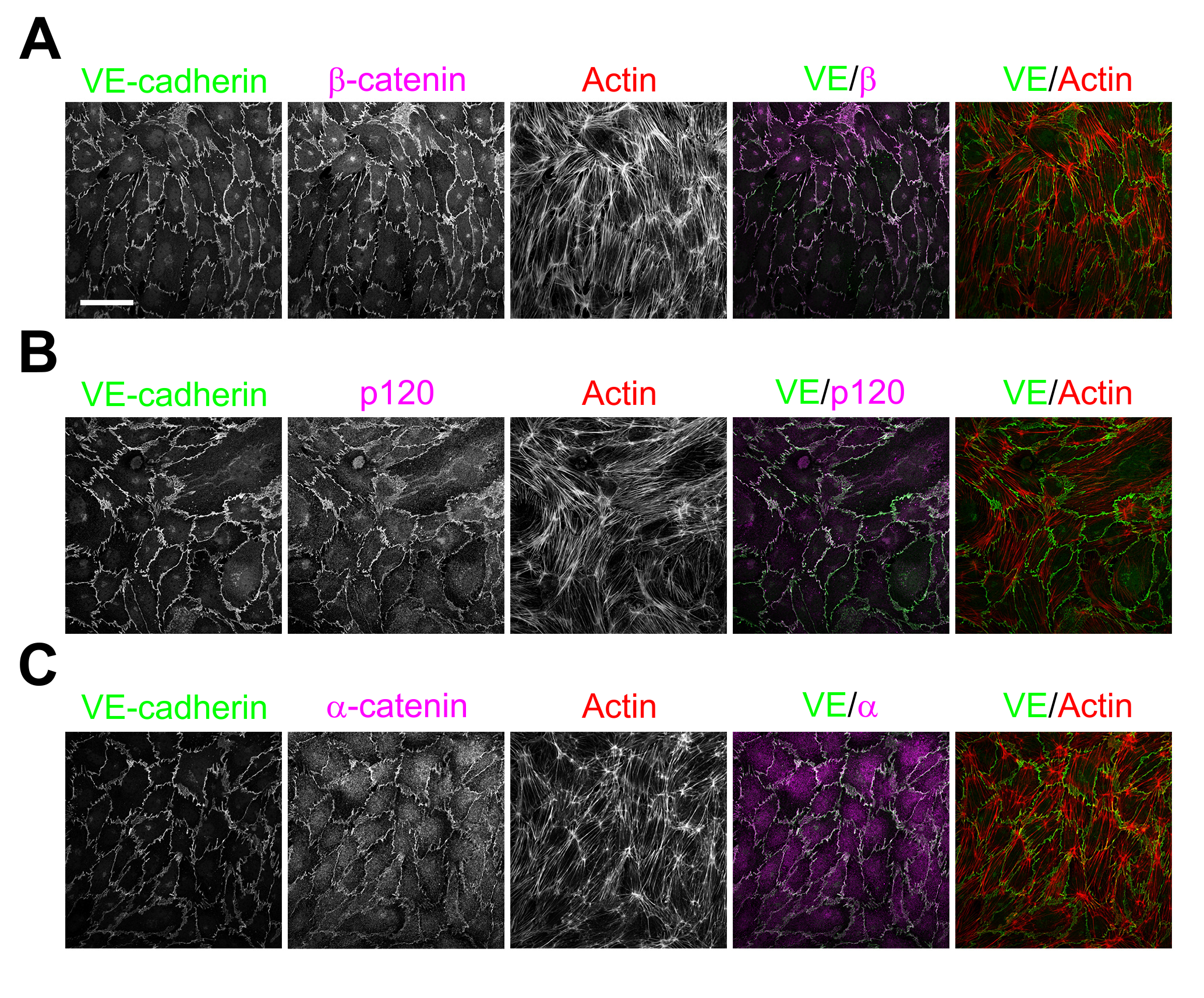

Supplement: S4 Fig — HUVEC were treated with TM5275 (50 μM) or solvent (DMSO 0.1%) for four hours and stained for VE-cadherin (green), actin and (A) β-catenin, (B) p120 catenin, or (C) α-catenin (red) (scale bar 50 μm). Junctions were disrupted and stress fibres were formed. For control see main text and Fig 3. (TIF) [file pone.0145684.s004.tif]

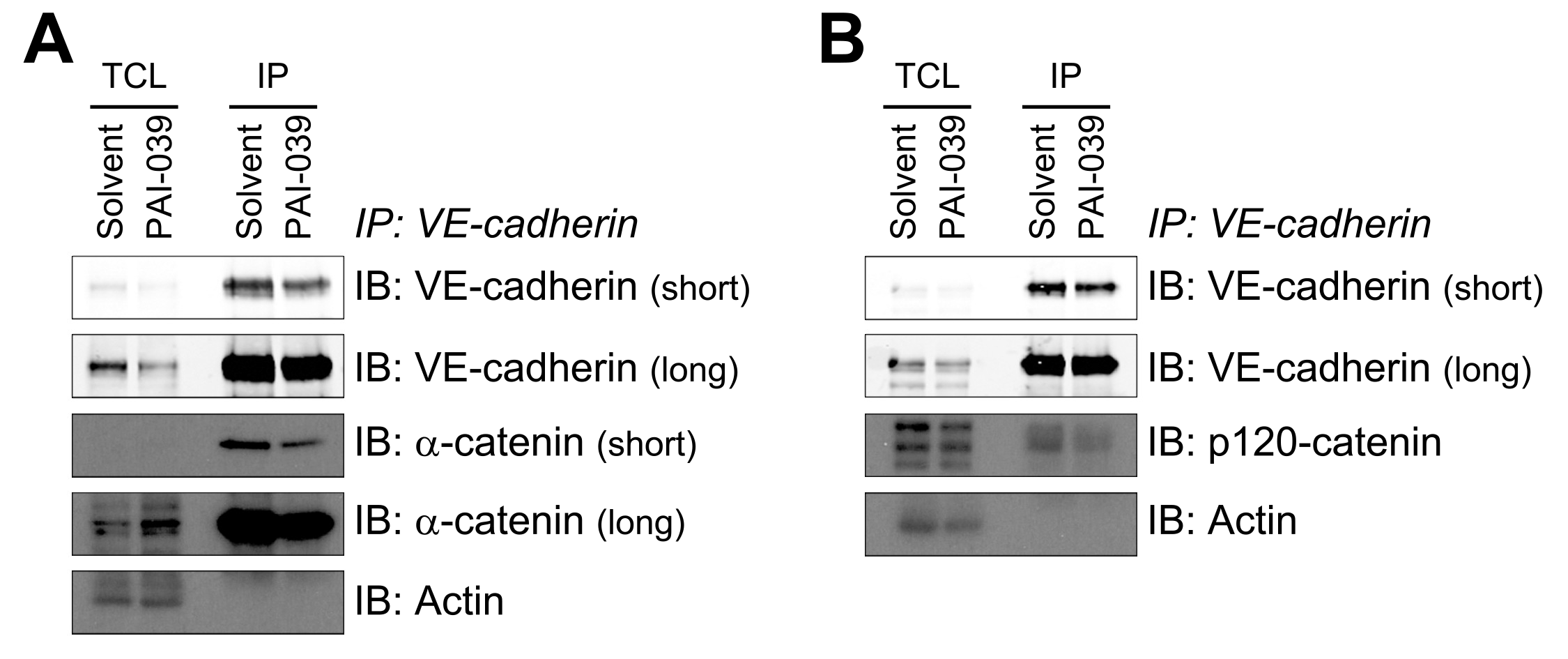

Supplement: S5 Fig — (A), (B), (C) FACS analysis of HUVEC treated with TM5275 (100 μM, control: DMSO 0.2%) or PAI-039 (25 μM, control: DMSO 0.1%) as indicated. PAI-1 inhibition did not affect VE-cadherin expression on HUVEC (n = 3, geometric mean fluorescence intensity + SEM, n.s. = non-significant). (D) Densitometric quantification of PAI-1 in conditioned media of HUVEC treated with PAI-039 as described in Fig 5B (n = 3, mean + SEM, n.s. = non-significant). (TIF) [file pone.0145684.s005.tif]

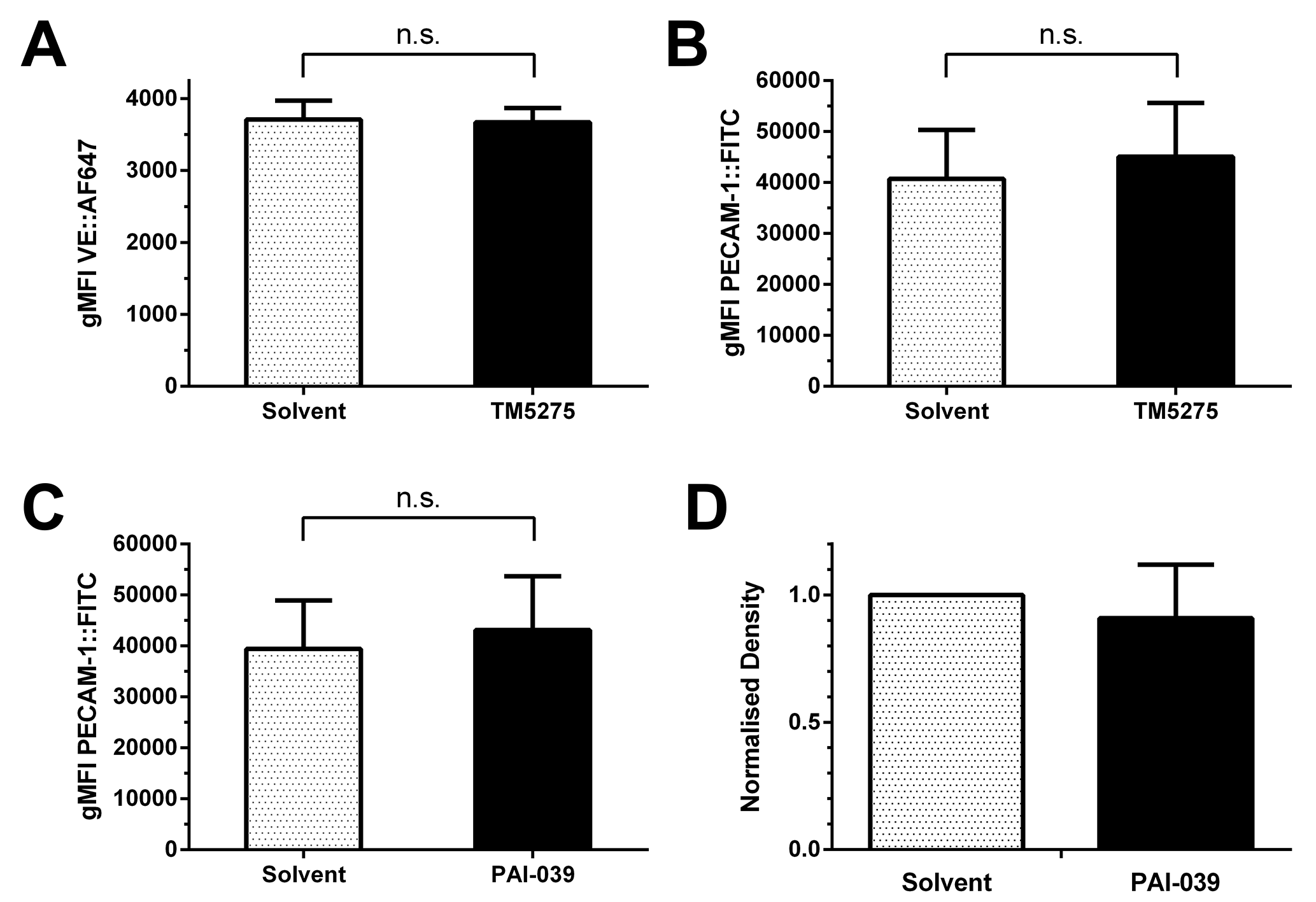

Supplement: S6 Fig — HUVEC were treated with PAI-039 (25 μM) or solvent (DMSO 0.1%) for four hours and total cell lysates (TCL) were subjected to immuno-coprecipitation (IP) of VE-cadherin. After SDS-PAGE proteins were blotted onto nitrocellulose membranes and the top half (> 70 kDa) first probed for (A) α-catenin or (B) p120 catenin (ECL) and then for VE-cadherin (Odyssey). The bottom half (< 70 kDa) was probed for β-actin. Short and long exposures are depicted here. (TIF) [file pone.0145684.s006.tif]
